# Supplementary material for: The rearing environment persistently modulates mouse phenotypes from the molecular to the behavioural level
Source: PLoS Biol. 2022 Oct 21;20(10):e3001837. doi: 10.1371/journal.pbio.3001837 (PMC9629646; doi:10.1371/journal.pbio.3001837)
Supplement: S2 Table — (PDF) [file pbio.3001837.s002.pdf]

S2 Table: ANOVA results for the effect of rearing facility (RF) and time point (TP) on α-diversity for males and females separately.

Sex	Response value	Covariate	Df	SumSq	MeanSq	F	p
Males	Shannon diversity	RF	4	0.6656	0.16639	1.44	0.2343
		TP	1	0.6791	0.67908	5.8769	0.0189*
		Residuals	51	5.8931			
	Chao1 richness	RF	4	32444	8110.9	3.9072	0.0076*
		TP	1	25840	25839.7	12.4477	8.959×10^{-04} *
		Residuals	51	105869	2075.9		
	Observed species richness	RF	4	29390	7347.6	4.6617	0.0028*
		TP	1	4636	4636	2.9413	0.0924
		Residuals	51	80384	1576.2		
	Pielou's evenness	RF	4	0.019379	0.004845	1.8004	0.1431
		TP	1	0.013766	0.013766	5.1158	0.0280*
		Residuals	51	0.137234	0.002691		
Females	Shannon diversity	RF	4	0.7651	0.191279	1.9047	0.1229
		TP	1	0.0059	0.005884	0.0586	0.8096
		Residuals	54	5.4228	0.100423		
	Chao1 richness	RF	4	21502	5375.6	2.9983	0.0263*
		TP	1	4302	4301.9	2.3995	0.1272
		Residuals	54	96815	1792.9		
	Observed species richness	RF	4	49045	12261.2	9.928	4.276×10^{-06} *
		TP	1	3604	3603.7	2.918	0.0933
		Residuals	54	66690	1235		
	Pielou's evenness	RF	4	0.012973	0.003243	1.4114	0.2426
		TP	1	0.000714	0.000714	0.3105	0.5797
		Residuals	54	0.124094	0.002298		
