# Supplementary material for: The rearing environment persistently modulates mouse phenotypes from the molecular to the behavioural level
Source: PLoS Biol. 2022 Oct 21;20(10):e3001837. doi: 10.1371/journal.pbio.3001837 (PMC9629646; doi:10.1371/journal.pbio.3001837)
Supplement: S3 Table — (PDF) [file pbio.3001837.s003.pdf]

S3 Table: PERMANOVA partitioning variation in microbiome microbial community composition (β-diversity) between rearing facilities (RF), for each timepoint (TP) and sex separately.

Sex	Time point (TP)	Covariate	Df	SumSq	R2	F	p
Males	TP1	RF	4	1.3324	0.2872	2.5179	9.999×10^{-05} *
		Residual	25	3.3073	0.7128		
		Total	29	4.6397	1		
	TP2	RF	4	0.7666	0.2038	1.4079	0.0004 *
		Residual	22	2.9947	0.7962		
		Total	26	3.7613	1		
Females	TP1	RF	4	1.107	0.2908	2.5628	9.999×10^{-05} *
		Residual	25	2.6996	0.7092		
		Total	29	3.8066	1		
	TP2	RF	4	0.6665	0.1699	1.2791	0.0444 *
		Residual	25	3.2567	0.8301		
		Total	29	3.9231	1		
