# Supplementary material for: The rearing environment persistently modulates mouse phenotypes from the molecular to the behavioural level
Source: PLoS Biol. 2022 Oct 21;20(10):e3001837. doi: 10.1371/journal.pbio.3001837 (PMC9629646; doi:10.1371/journal.pbio.3001837)
Supplement: S4 Table — The effect of rearing environment on body weight was evaluated at 3 TPs throughout the study: right before killing within each RF (PND 56; TP1), after the acclimatisation period in the testing facility (PND 75), and at the end of the experiment (PND 102; TP2). Linear models were used to analyse data collected on mouse body weight at PND 56 (TP1) within each RF for males and females (a). RF, litter size, and sex ratio at weaning were used as predictor variables. Linear mixed effect models with the same list of predictor variables as fixed effects were used for the body weight data collected in the testing facility for males and females (b). Cage identification number (cage ID) in the testing facility was used as a random factor. (a) Linear regression model outcomes for body weight data collected in each RF; (b) Linear mixed effect model with type III ANOVA with Satterthwaite’s approximation for body weight data collected at the testing facility. PND, postnatal day; RF, rearing facility; TP, time point. (PDF) [file pbio.3001837.s004.pdf]

S4 Table: Phenotypic variation in body weight of mice is induced by common differences between the rearing conditions in different facilities.

The effect of rearing environment on body weight was evaluated at three time points throughout the study: right before euthanasia within each rearing facility (PND 56; TP1), after the acclimatization period in the testing facility (PND 75) and at the end of the experiment (PND 102; TP2).

Linear models were used to analyze data collected on mouse body weight at PND 56 (TP1) within each rearing facility for males and females (a)**.** Rearing facility, litter size and sex ratio at weaning were used as predictor variables.

Linear mixed effect models with the same list of predictor variables as fixed effects were used for the body weight data collected in the testing facility for males and females (b). Cage identification number (cage ID) in the testing facility was used as a random factor.

a) Linear regression model outcomes for body weight data collected in each rearing facility.

Sex	Variables	Df	Sum Sq	Mean Sq	F value	p
Males	Rearing Facility	4	62.619	15.6525	9.7496	9.185×10^{-05}*
	Litter size at weaning	1	0.577	0.5769	0.3593	0.5547^{ns}
	Sex ratio at weaning	1	0.014	0.0141	0.0088	0.9263^{ns}
	Residuals	23	36.925	1.6055		
Females	Rearing Facility	4	28.6847	7.1712	8.4225	2.447×10^{-04} *
	Litter size at weaning	1	4.1711	4.1411	4.8989	0.0371^{ns}
	Sex ratio at weaning	1	0.371	0.371	0.4357	0.5158^{ns}
	Residuals	23	19.583	0.8514		

b) Linear mixed effect model with type III ANOVA with Satterthwaite's approximation for body weight data collected at the testing facility.

Sex	PND	Fixed factors	Sum Sq	Mean Sq	num Df	den Df	F value	p
**Males**	BW after acclimatization period (PND 75)	Rearing Facility	20.5856	5.1464	4	52	5.1178	0.0015*
		Litter size at weaning	1.0737	1.0737	1	52	1.0677	0.3062 ns
		Sex ratio at weaning	0.5034	0.5034	1	52	0.5006	0.4824 ns
		Number of cage mates after weaning	0.1953	0.1953	1	52	0.1943	0.6612 ns
		Random factor: Cage ID in the testing lab						
		REML criterion at convergence: 399.7						
		marginal R2 0.3071549; conditional R2 0.6517345						
		**Fixed factors**	**Sum Sq**	**Mean Sq**	**num Df**	**den Df**	**F value**	**p**
	BW at the end of the experiment (PND 102; TP2)	Rearing Facility	19.1941	4.7985	4	52	3.5935	0.0116*
		Litter size at weaning	0.2587	0.2587	1	52	0.1938	0.6616 ns
		Sex ratio at weaning	0.8734	0.8734	1	52	0.6541	0.4223 ns
		Number of cage mates after weaning	1.0087	1.0087	1	52	0.7554	0.3888 ns
		Random factor: Cage ID in the testing lab						
		REML criterion at convergence: 429.4						
		marginal R2 0.2106322; conditional R2 0.5912489						
**Females**	**PND**	**Fixed factors**	**Sum Sq**	**Mean Sq**	**num Df**	**den Df**	**F value**	**p**
	BW after acclimatization period (PND 75)	Rearing Facility	18.1681	4.542	4	52	9.0795	1.221×10^{-05}*
		Litter size at weaning	0.1548	0.1548	1	52	0.3095	0.5804 ns
		Sex ratio at weaning	0.074	0.074	1	52	0.1479	0.7022 ns
		Number of cage mates after weaning	0.0361	0.0361	1	52	0.0722	0.7892 ns
		Random factor: Cage ID in the testing lab						
		REML criterion at convergence: 399.7						
		marginal R2 0.3071549; conditional R2 0.6517345						
		**Fixed factors**	**Sum Sq**	**Mean Sq**	**num Df**	**den Df**	**F value**	**p**
	BW at the end of the experiment (PND 102; TP2)	Rearing Facility	17.5852	4.3963	4	52	6.9142	1.533×10^{-04} *
		Litter size at weaning	0.0314	0.0314	1	52	0.0494	0.8250 ns
		Sex ratio at weaning	0.0118	0.0118	1	52	0.0186	0.8921 ns
		Number of cage mates after weaning	0.2106	0.2106	1	52	0.3312	0.5674 ns
		Random factor: Cage ID in the testing lab						
		REML criterion at convergence: 429.4						
		marginal R2 0.2106322; conditional R2 0.5912489						
