# Supplementary material for: The rearing environment persistently modulates mouse phenotypes from the molecular to the behavioural level
Source: PLoS Biol. 2022 Oct 21;20(10):e3001837. doi: 10.1371/journal.pbio.3001837 (PMC9629646; doi:10.1371/journal.pbio.3001837)
Supplement: S5 Table — MANOVA outcomes and statistics for males and females. The RF was defined as a main independent (predictor) variable. Additional covariates were included in the model based on the published evidence, which suggests that they might affect behavioral phenotype. List of the nuisance variables includes: litter size at weaning [1,2], sex ratio at weaning [3,4], and number of cage mates after weaning [5]. In addition, we included the stage of oestrous cycle at the time of testing (OF ECS and LDB ECS) in females, because of its effects on behaviour [6–8]. The results were confirmed by comparing the Pillai’s Trace outcome with outcomes of 3 different test statistics. The proportion of variation in behaviour of mice, which is solely attributed to differences in rearing environments, was calculated by dividing the Pillai’s trace by the degrees of freedom. MANOVA outcomes and statistics for behavioural parameters. LDB, light–dark box; MANOVA, multivariate analysis of variance; OF, open field; RF, rearing facility. (PDF) [file pbio.3001837.s005.pdf]

S5 Table: Phenotypic variation in behavior of mice is modified by common differences between the rearing conditions in different facilities.

MANOVA outcomes and statistics for males and females. The rearing facility was defined as a main independent (predictor) variable. Additional covariates were included in the model based on the published evidence, which suggests that they might affect behavioral phenotype. List of the nuisance variables includes: litter size at weaning (1,2), sex ratio at weaning (3,4) and number of cage mates after weaning (5). In addition, we included the stage of oestrous cycle at the time of testing (OF ECS and LDB ECS) in females, because of its effects on behaviour (6–8). The results were confirmed by comparing the Pillai’s Trace outcome with outcomes of three different test statistics. The proportion of variation in behaviour of mice, which is solely attributed to differences in rearing environments, was calculated by dividing the Pillai's trace by the degrees of freedom.

MANOVA outcomes and statistics for behavioural parameters.

Sex	Variables	Df	Test statistic	approx. F	num Df	den Df	p
Males	Rearing Facility	4	0.86346	2.29409	24	200	9.85×10^{-04} *
	Litter size at weaning	1	0.14918	1.37344	6	47	0.2450 ^{ns}
	Sex ratio at weaning	1	0.08369	0.71544	6	47	0.6390 ^{ns}
	Number of cage mates after weaning	1	0.1701	1.60553	6	47	0.1667 ^{ns}

Sex	Rearing Facility	Test statistic	approx. F	Df (F)	p
Males	Pillai's Trace	0.86346	2.29409	24, 200	9.85×10^{-04} *
	Wilk's Lambda	0.35883	2.35026	24, 165	8.72×10^{-04} *
	Hotelling-Lawley Trace	1.23437	2.34015	24, 182	8.29×10^{-04} *
	Roy's Largest Root	0.52668	4.389	6, 1950	0.0012 ^{ns}

Sex	Variables	Df	Test statistic	approx. F	num Df	den Df	p
Females	Rearing Facility	4	0.74167	1.821	24	192	0.0144 *
	Litter size at weaning	1	0.17471	1.5877	6	45	0.1728 ^{ns}
	Sex ratio at weaning	1	0.05987	0.4776	6	45	0.8214 ^{ns}
	Number of cage mates after weaning	1	0.06705	0.539	6	45	0.7757 ^{ns}
	OF ECS	1	0.40478	5.1005	6	45	0.0005*
	LDB ECS	1	0.50042	7.5127	6	45	1.30×10^{-05} *

Sex	Rearing Facility	Test statistic	approx. F	Df (F)	p
Females	Pillai's Trace	0.74167	1.821	24, 192	0.0144*
	Wilk's Lambda	0.4207	1.5877	24, 158	0.0132*
	Hotelling-Lawley Trace	1.02484	1.8575	24, 174	0.0125*
	Roy's Largest Root	0.53902	4.3122	6, 48	0.0015*

1. Tanaka T. Effects of litter size on behavioral development in mice. Reproductive Toxicology. 1998 Nov;12(6):613–7.
2. Salari AA, Samadi H, Homberg JR, Kosari-Nasab M. Small litter size impairs spatial memory and increases anxiety- like behavior in a strain-dependent manner in male mice. Sci Rep. 2018 Dec;8(1):11281.
3. Namikas J, Wehmer F. Gender composition of the litter affects behavior of male mice. Behav Biol. 1978 Jun;23(2):219–24.
4. Laviola G, Alleva E. Sibling effects on the behavior of infant mouse litters (Mus domesticus). Journal of Comparative Psychology. 1995;109(1):68–75.
5. Horii Y, Nagasawa T, Sakakibara H, Takahashi A, Tanave A, Matsumoto Y, et al. Hierarchy in the home cage affects behaviour and gene expression in group-housed C57BL/6 male mice. Sci Rep. 2017 Dec;7(1):6991.
6. Jaric I, Rocks D, Greally JM, Suzuki M, Kundakovic M. Chromatin organization in the female mouse brain fluctuates across the oestrous cycle. Nat Commun. 2019 Dec;10(1):2851.
7. Jaric I, Rocks D, Cham H, Herchek A, Kundakovic M. Sex and Estrous Cycle Effects on Anxiety- and Depression-Related Phenotypes in a Two-Hit Developmental Stress Model. Front Mol Neurosci. 2019;12:74.
8. Chen W, Shields J, Huang W, King JA. Female fear: influence of estrus cycle on behavioral response and neuronal activation. Behav Brain Res. 2009 Jul 19;201(1):8–13.
