# Supplementary material for: The rearing environment persistently modulates mouse phenotypes from the molecular to the behavioural level
Source: PLoS Biol. 2022 Oct 21;20(10):e3001837. doi: 10.1371/journal.pbio.3001837 (PMC9629646; doi:10.1371/journal.pbio.3001837)
Supplement: S6 Table — The rearing facility was defined as a grouping variable, while the 6 behavioral measures served as predictor variables. (OF = open field test, LDB = light–dark box test). (PDF) [file pbio.3001837.s006.pdf]

S6_Table: Loadings of a LDA for male and female behaviour. The rearing facility was defined as a grouping variable, while the six behavioral measures served as predictor variables. (OF=open field test, LDB=light-dark box test).

Sex	Test	LD1	LD2	LD3	LD4
Males	OF distance	0.854	-0.3871	-0.7171	-0.1959
	OF time center	-0.4286	0.3425	0.0036	-0.7893
	OF latency	-0.5578	0.5945	-0.7424	-0.5394
	LDB time light	0.7345	0.8493	0.1656	-0.2777
	LDB entries into light	0.1125	0.6679	-0.1703	0.7091
	LDB latency	0.277	0.1903	0.0283	-0.1056
**Sex**	**Test**	**LD1**	**LD2**	**LD3**	**LD4**
Females	OF distance	0.8961	-0.204	0.8567	-0.0828
	OF time center	-0.6191	-1.0171	0.2796	0.2336
	OF latency	-0.2851	-0.0715	0.3807	-0.4671
	LDB time light	-0.4893	-0.4805	-0.1006	-0.4322
	LDB entries into light	0.1472	-0.2259	-1.3008	0.0167
	LDB latency	-0.2132	0.0024	-0.1594	0.8519
