# Supplementary material for: The rearing environment persistently modulates mouse phenotypes from the molecular to the behavioural level
Source: PLoS Biol. 2022 Oct 21;20(10):e3001837. doi: 10.1371/journal.pbio.3001837 (PMC9629646; doi:10.1371/journal.pbio.3001837)
Supplement: S7 Table — (PDF) [file pbio.3001837.s007.pdf]

S7 Table: Classification based on LDA of behaviour of males and females.

Males		Predicted Rearing Facility (RF)				
		RF 1	RF 2	RF 3	RF 4	RF 5
Actual Rearing Facility (RF)	RF 1	**7**	1	2	1	1
	RF 2	0	**7**	2	2	1
	RF 3	1	1	**9**	1	0
	RF 4	2	2	1	**7**	0
	RF 5	2	2	1	2	**5**

Females		Predicted Rearing Facility (RF)				
		RF 1	RF 2	RF 3	RF 4	RF 5
Actual Rearing Facility (RF)	RF 1	**6**	1	1	3	1
	RF 2	1	**6**	2	1	2
	RF 3	1	0	**8**	2	1
	RF 4	2	2	2	**6**	0
	RF 5	1	1	2	2	**6**
