# Supplementary material for: The rearing environment persistently modulates mouse phenotypes from the molecular to the behavioural level
Source: PLoS Biol. 2022 Oct 21;20(10):e3001837. doi: 10.1371/journal.pbio.3001837 (PMC9629646; doi:10.1371/journal.pbio.3001837)
Supplement: S8 Table — Statistical outcomes of plasma corticosterone measures for males and females. We applied linear models, with the covariates rearing facility, litter size at weaning, sex ratio at weaning, and number of cage mates after weaning. In females, the stage of oestrous cycle on the testing day was also included, and data were grouped into high- and low-oestrogen state. (PDF) [file pbio.3001837.s008.pdf]

S8 Table: Phenotypic differences of the HPA stress profile in mice cannot be explained by common differences between the rearing conditions in different facilities.

Statistical outcomes of plasma corticosterone measures for males and females. We applied linear models, with the covariates rearing facility, litter size at weaning, sex ratio at weaning and number of cage mates after weaning. In females, the stage of oestrous cycle on the testing day was also included, and data were grouped into high- and low-oestrogen state.

Sex	Value	Variables	Df	Sum Sq	Mean Sq	F value	p
Males	Basal	Rearing Facility	4	1089.3	272.3	0.7134	0.5865 ns
		Litter size at weaning	1	2820.5	2820.5	7.3891	0.0089*
		Sex ratio at weaning	1	83.2	83.2	0.2179	0.6426 ns
		Number of cage mates after weaning	1	8152.7	8152.7	21.3581	2.549×10^{-05}*
		Residuals	52	19849.2	381.7		
	Response	Rearing Facility	4	15273	3818	0.6879	0.6036 ns
		Litter size at weaning	1	475	475	0.0856	0.7710 ns
		Sex ratio at weaning	1	598	598	0.1078	0.7440 ns
		Number of cage mates after weaning	1	39812	39812	7.1723	0.0099*
		Residuals	52	288646	5551		
	Recovery	Rearing Facility	4	29750	7437.6	2.9757	0.0275 ns
		Litter size at weaning	1	27	27.4	0.011	0.9170 ns
		Sex ratio at weaning	1	1137	1136.8	0.4548	0.5030 ns
		Number of cage mates after weaning	1	1277	1276.8	0.5109	0.4780 ns
		Residuals	52	129971	2499.4		
**Sex**	**Value**	**Variables**	**Df**	**Sum Sq**	**Mean Sq**	**F value**	**p**
Females	Basal	Rearing Facility	4	3128	782	0.6902	0.6021 ns
		Litter size at weaning	1	1296	1295.5	1.1433	0.2900 ns
		Sex ratio at weaning	1	46	46.1	0.0407	0.8409 ns
		Number of cage mates after weaning	1	73	73	0.0644	0.8006
		CORT ESC	1	26958	26957.9	23.7911	1.093×10^{-05}*
		Residuals	51	57788	1131.1		
	Response	Rearing Facility	4	39010	9752.5	1.7054	0.1632 ns
		Litter size at weaning	1	17	17.5	0.0031	0.9561 ns
		Sex ratio at weaning	1	471	471.2	0.0824	0.7752 ns
		Number of cage mates after weaning	1	4331	4330.6	0.7573	0.3883 ns
		CORT ESC	1	1542	1541.9	0.2696	0.6058 ns
		Residuals	51	288646	5551		
	Recovery	Rearing Facility	4	41128	10281.9	1.2577	0.2987 ns
		Litter size at weaning	1	18892	18892	2.3109	0.1346 ns
		Sex ratio at weaning	1	1943	1942.9	0.2377	0.6280 ns
		Number of cage mates after weaning	1	2	1.6	0.0002	0.9891 ns
		CORT ESC	1	7510	7510.1	0.9186	0.3424 ns
		Residuals	51	416938	8175.3		
