# Supplementary material for: The rearing environment persistently modulates mouse phenotypes from the molecular to the behavioural level
Source: PLoS Biol. 2022 Oct 21;20(10):e3001837. doi: 10.1371/journal.pbio.3001837 (PMC9629646; doi:10.1371/journal.pbio.3001837)
Supplement: S9 Table — Statistical outcomes for relative adrenal gland weight data collected at the testing laboratory (TP1; PND 56) for males and females. Linear mixed effect models were used with rearing facility, litter size at weaning, sex ratio at weaning, and number of cage mates after weaning as fixed effect, while the cage ID in the testing facility was defined as a random factor. Linear mixed effect model with type III ANOVA with Satterthwaite’s approximation for relative adrenal gland weight for males. (PDF) [file pbio.3001837.s009.pdf]

S9 Table: Phenotypic variation in relative adrenal weight of mice is induced by common differences between the rearing conditions in different facilities.

Statistical outcomes for relative adrenal gland weight data collected at the testing laboratory (TP1; PND 56) for males and females. Linear mixed effect models were used with rearing facility, litter size at weaning, sex ratio at weaning and number of cage mates after weaning as fixed effect, while the cage ID in the testing facility was defined as a random factor.

Linear mixed effect model with type III ANOVA with Satterthwaite's approximation for relative adrenal gland weight for males.

Sex	Fixed factors	Sum Sq	Mean Sq	num Df	den Df	F value	p
Males	Rearing Facility	$6.06e^{-05}$	1.5138×10^{-05}	4	52	5.4086	1.02×10^{-03} *
	Litter size at weaning	$2.48e^{-06}$	2.4788×10^{-06}	1	52	0.8857	0.3510^{ns}
	Sex ratio at weaning	$1.26e^{-06}$	1.2639×10^{-06}	1	52	0.4516	0.5045^{ns}
	Number of cage mates after weaning	$9.61e^{-07}$	9.6140×10^{-07}	1	52	0.3435	0.5603^{ns}
	Random factor: Cage ID in the testing lab						
	REML criterion at convergence: -1032.6						
	marginal R2 0.2589189; conditional R2 0.3630702						
Females	Rearing Facility	$5.56e^{-05}$	$1.39e^{-05}$	4	52	0.9054	0.4677*
	Litter size at weaning	$6.99e^{-07}$	$6.99e^{-06}$	1	52	0.0456	0.8318^{ns}
	Sex ratio at weaning	$3.50e^{-05}$	$3.50e^{-06}$	1	52	2.2827	0.1369^{ns}
	Number of cage mates after weaning	$1.02e^{-05}$	$1.02e^{-05}$	1	52	0.6636	0.4190^{ns}
	Random factor: Cage ID in the testing lab						
	REML criterion at convergence: -1032.6						
	marginal R2 0.2589189; conditional R2 0.3630702						
