# Supplementary material for: The rearing environment persistently modulates mouse phenotypes from the molecular to the behavioural level
Source: PLoS Biol. 2022 Oct 21;20(10):e3001837. doi: 10.1371/journal.pbio.3001837 (PMC9629646; doi:10.1371/journal.pbio.3001837)
Supplement: S10 Table — Manhattan distance between all samples was used as input. (PDF) [file pbio.3001837.s010.pdf]

S10 Table: PERMANOVA partitioning variation in chromatin accessibility profile between rearing facilities and processing batches. Manhattan distance between all samples was used as input.

Chromatin accessibility profile	**Rearing Laboratory**		**Processing Batch**	
	Sum Sq	p	Sum Sq	p
all sites TP1	55.33	$1.00e^{-04}$*	11.13	0.1431
all sites TP2	36.79	$1.00e^{-04}$*	12.93	0.3862
open chromatin sites TP1	77.53	$1.00e^{-04}$*	10.01	0.0063
open chromatin sites TP2	70.91	$1.00e^{-04}$*	6.5	0.3343
closed chromatin sites TP1	48.24	$1.00e^{-04}$*	11.83	0.1998
closed chromatin sites TP2	28.44	$1.00e^{-04}$*	14.37	0.4184
