# Supplementary material for: The rearing environment persistently modulates mouse phenotypes from the molecular to the behavioural level
Source: PLoS Biol. 2022 Oct 21;20(10):e3001837. doi: 10.1371/journal.pbio.3001837 (PMC9629646; doi:10.1371/journal.pbio.3001837)
Supplement: S11 Table — (PDF) [file pbio.3001837.s011.pdf]

S11 Table: The litter size, the litter sex ratio and number of pups weaned from each litter across rearing facilities.

Rearing facility (RF)	Mother cage ID	Litter size	No. of pups born		No. of pups weaned	
			M	F	M	F
RF1	1H1	7	3	4	3	3
	1H2	9	5	4	3	3
	1H3	7	5	2	3	2
	1H4	6	3	3	3	3
	1H5	7	2	5	NA	3
	1H6	6	4	2	3	2
	1H8	7	4	3	NA	3
	1H9	3	0	3	NA	3
	1H10	8	4	4	3	3
	1H11	5	4	1	3	NA
	1H12	5	4	1	3	NA
	1H13	7	4	3	3	3
	1H15	5	3	2	3	NA
	1H16	8	4	4	3	3
	1H17	7	5	2	3	NA
	1H18	7	5	2	NA	2
RF2	2H1	8	2	6	2	3
	2H2	9	5	4	2	3
	2H3	8	4	4	3	3
	2H4	7	4	3	3	3
	2H6	6	3	3	3	3
	2H7	5	2	3	NA	3
	2H8	8	5	3	3	3
	2H9	6	4	2	3	NA
	2H10	6	4	2	3	NA
	2H12	7	4	3	3	3
	2H13	7	3	4	3	3
	2H14	5	2	3	NA	3
	2H15	8	5	3	3	3
	2H16	4	1	3	NA	3
	2H18	5	2	3	2	NA
RF3	B1	4	1	3	NA	3
	B2	7	1	3	NA	3
	B3	3	2	1	2	NA
	B4	7	3	4	3	3
	B5	7	3	4	3	3
	B6	4	2	2	2	2
	B7	9	5	4	3	3
	B8	6	4	2	3	2
	B9	6	4	2	3	2
	B12	9	7	2	3	2
	B13	3	3	0	2	NA
	B14	6	3	3	2	2
	B15	4	2	2	2	2
	B18	6	2	4	2	3

Rearing facility (RF)	Mother cage ID	Litter size	No. of pups born		No. of pups weaned	
			M	F	M	F
RF4	M1	8	5	3	3	NA
	M2	9	3	6	3	3
	M3	8	2	6	NA	3
	M5	10	3	7	NA	3
	M6	4	2	2	2	NA
	M7	5	2	3	NA	3
	M9	7	3	4	3	3
	M10	8	4	4	3	3
	M11	10	6	4	3	3
	M12	8	4	4	3	NA
	M13	7	1	6	NA	3
	M14	8	3	5	3	3
	M15	7	4	3	3	3
	M16	8	4	4	3	NA
	M17	9	3	6	3	3
	M18	7	4	3	3	3
RF5	Z1	6	5	1	3	NA
	Z2	7	3	4	3	3
	Z3	4	3	1	3	NA
	Z4	7	5	2	3	NA
	Z6	5	2	3	NA	3
	Z7	8	2	6	NA	3
	Z8	6	2	4	2	3
	Z9	7	7	0	3	NA
	Z10	8	4	4	3	3
	Z11	7	4	3	3	3
	Z12	6	5	1	3	NA
	Z13	8	5	3	3	3
	Z14	4	2	2	NA	2
	Z15	9	3	6	NA	3
	Z16	9	5	4	3	3
	Z17	6	1	5	NA	3
	Z18	6	4	2	3	2
