# Supplementary material for: The rearing environment persistently modulates mouse phenotypes from the molecular to the behavioural level
Source: PLoS Biol. 2022 Oct 21;20(10):e3001837. doi: 10.1371/journal.pbio.3001837 (PMC9629646; doi:10.1371/journal.pbio.3001837)
Supplement: S12 Table — Several highly correlated variables were removed before a multivariate analysis. Only variables with moderated correlations amongst each other were used. (a) Pearson product moment correlations for behavioural measures for males. Mean absolute correlation coefficient for males was 0.20. (b) Pearson product moment correlations for behavioural measures for females. Mean absolute correlation coefficient for females: 0.29. (PDF) [file pbio.3001837.s012.pdf]

S12 Table: Pearson product moment correlations for behavioural measures for males **(a)** and females **(b)**. Several highly correlated variables were removed before a multi-variate analysis. Only variables with moderated correlations amongst each other were used.

a) Pearson product moment correlations for behavioural measures for males. Mean absolute correlation coefficient for males was 0.20.

MALES	OF distance	OF velocity	OF time centre	OF entries centre	OF latency	LDB distance	LDB velocity	LDB time light	LDB entries	LDB latency
**OF distance**		1	0.27	0.83	-0.17	0.55	0.46	0.3	0.23	0.07
OF velocity			0.27	0.83	-0.17	0.55	0.46	0.3	0.23	0.07
**OF time centre**				0.61	-0.45	0.19	0	0.23	0.11	-0.04
OF entries centre					-0.27	0.51	0.38	0.31	0.2	0.05
**OF latency**						0.01	0.09	-0.08	0	0.11
LDB distance							0.6	0.71	0.51	-0.2
LDB velocity								-0.12	0.38	-0.01
**LDB time light**									0.28	-0.2
**LDB entries**										-0.41
**LDB latency**										

b) Pearson product moment correlations for behavioural measures for females. Mean absolute correlation coefficient for females: 0.29.

FEMALES	OF distance	OF velocity	OF time centre	OF entries centre	OF latency	LDB distance	LDB velocity	LDB time light	LDB entries	LDB latency
**OF distance**		1	0.46	0.89	-0.17	0.56	0.48	0.25	0.56	-0.27
OF velocity			0.46	0.89	-0.17	0.56	0.48	0.25	0.56	-0.27
**OF time centre**				0.71	-0.2	0.33	0.19	0.25	0.41	-0.24
OF entries centre					-0.22	0.48	0.41	0.22	0.48	-0.29
**OF latency**						-0.1	-0.02	-0.13	0.01	0.11
LDB distance							0.61	0.66	0.65	-0.41
LDB velocity								-0.18	0.4	-0.13
**LDB time light**									0.41	-0.38
**LDB entries**										-0.49
**LDB latency**										
