# Supplementary material for: The rearing environment persistently modulates mouse phenotypes from the molecular to the behavioural level
Source: PLoS Biol. 2022 Oct 21;20(10):e3001837. doi: 10.1371/journal.pbio.3001837 (PMC9629646; doi:10.1371/journal.pbio.3001837)
Supplement: S1 Fig — BT mice, mice used for behavioural testing; GD, gestational day; LDB, light–dark box test; MA mice, mice that were not behaviourally tested and used for chromatin profiling and gut microbiome composition analysis; OF, open field test; PND, postnatal day; SRT,: stress reactivity test; TP, time point. (PDF) [file pbio.3001837.s013.pdf]

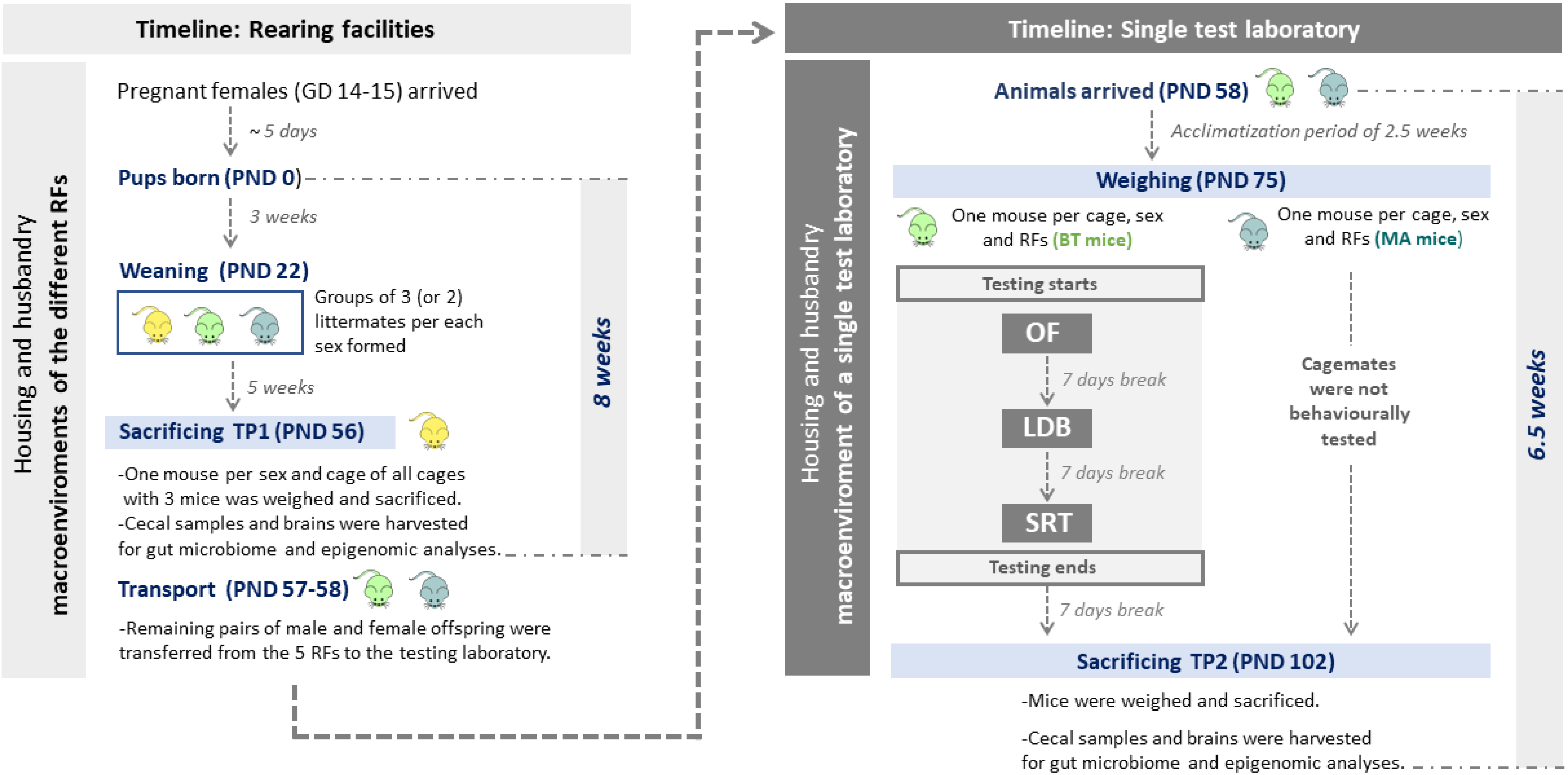

S1 Figure: Timeline of the study. GD: gestational day: PND: postnatal day; TP: time point; BT mice: mice used for behavioural testing mice; MA mice: mice that were not behaviourally tested and used for chromatin profiling and gut microbiome composition analysis. OF: open field test; LDB: light-dark box test; SRT: Stress reactivity test.
