# Supplementary material for: The rearing environment persistently modulates mouse phenotypes from the molecular to the behavioural level
Source: PLoS Biol. 2022 Oct 21;20(10):e3001837. doi: 10.1371/journal.pbio.3001837 (PMC9629646; doi:10.1371/journal.pbio.3001837)
Supplement: S2 Fig — (a) Dendrogram representing relationship between samples from mice at TP1 following hierarchical clustering (average linkage). Horizontal bars below the dendrogram represent sample identity in relation to rearing facility (first bar) or diet supplier (second bar). (b) Mean abundance of amplicon sequence variants (ASVs) identified as differentially abundant between the 2 diet suppliers aggregated by phylum (left) and order (right). Significance symbols represent results from Wilcoxon paired-rank test. The raw data underlying this figure are available in the Figshare repository https://doi.org/10.6084/m9.figshare.21087688. The 16S rRNA gene sequencing data are available from the European Nucleotide Archive (ENA) under accession number PRJEB49361. (PDF) [file pbio.3001837.s014.pdf]

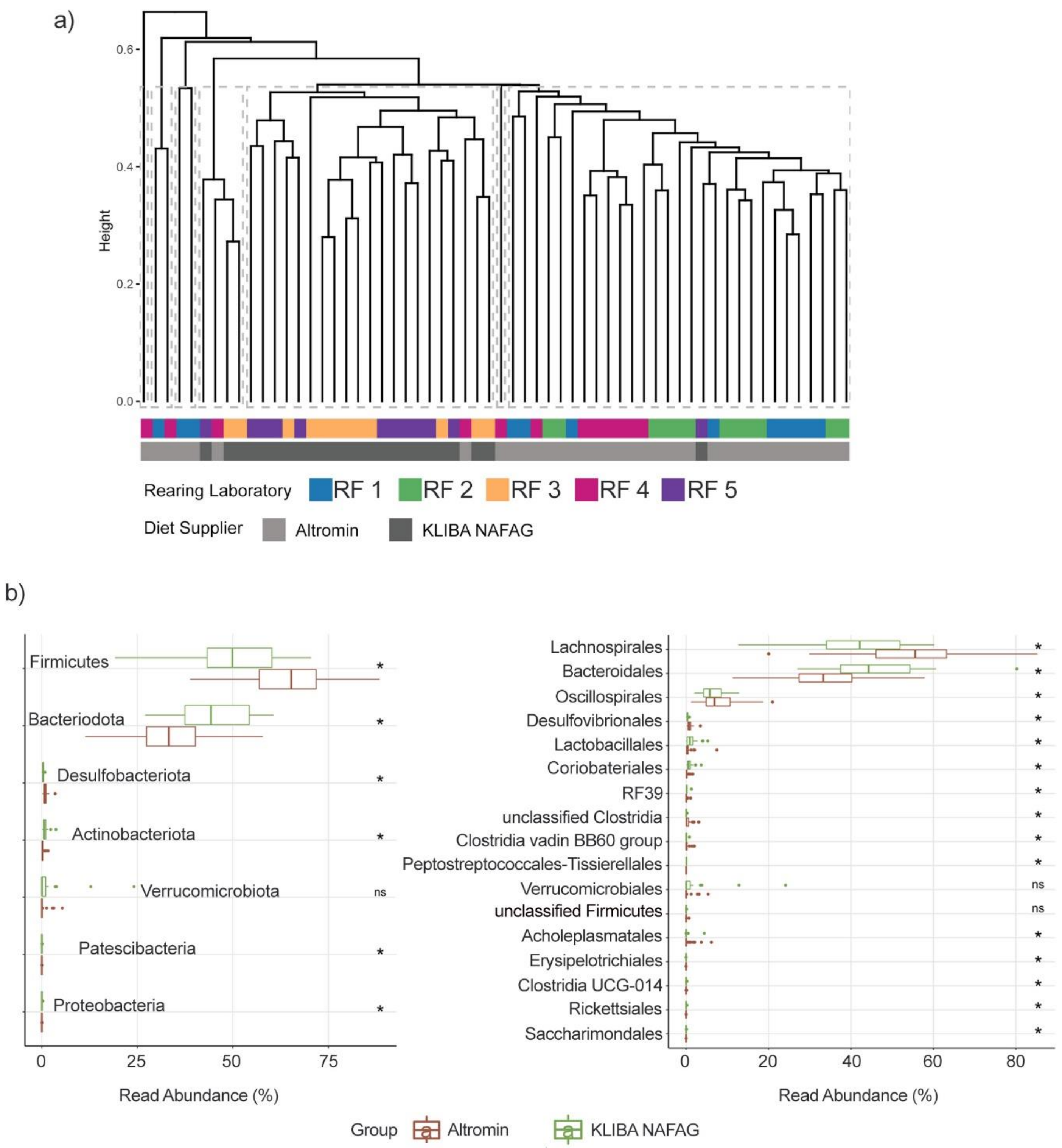

S2 Figure: Diet suppliers: Differentially abundant taxa. a) Dendrogram representing relationship between samples from mice at TP1 following hierarchical clustering (average linkage). Horizontal bars below the dendrogram represent sample identity in relation to rearing facility (first bar) or diet supplier (second bar). **b)** Mean abundance of amplicon sequence variants (ASVs) identified as differentially abundant between the two diet suppliers aggregated by phylum (left) and order (right). Significance symbols represent results from Wilcoxon paired-rank test. The raw data underlying this figure are available in the Figshare repository https://doi.org/10.6084/m9.figshare.21087688. The 16S rRNA gene sequencing data available from the European Nucleotide Archive (ENA) under accession number PRJEB49361.
