# Supplementary material for: The rearing environment persistently modulates mouse phenotypes from the molecular to the behavioural level
Source: PLoS Biol. 2022 Oct 21;20(10):e3001837. doi: 10.1371/journal.pbio.3001837 (PMC9629646; doi:10.1371/journal.pbio.3001837)
Supplement: S3 Fig — Results of the open field (a, b) and light-dark box (c) tests are presented in females depending on the oestrous cycle stage determined immediately after behavioral testing. There was a significant effect of oestrogen status on time spent in the centre (b) and time spent in the light compartment (c) with high-oestrogenic females showing marginally higher activity than low-oestrogenic females. The raw data underlying this figure are available in the Figshare repository https://doi.org/10.6084/m9.figshare.21087718. (PDF) [file pbio.3001837.s015.pdf]

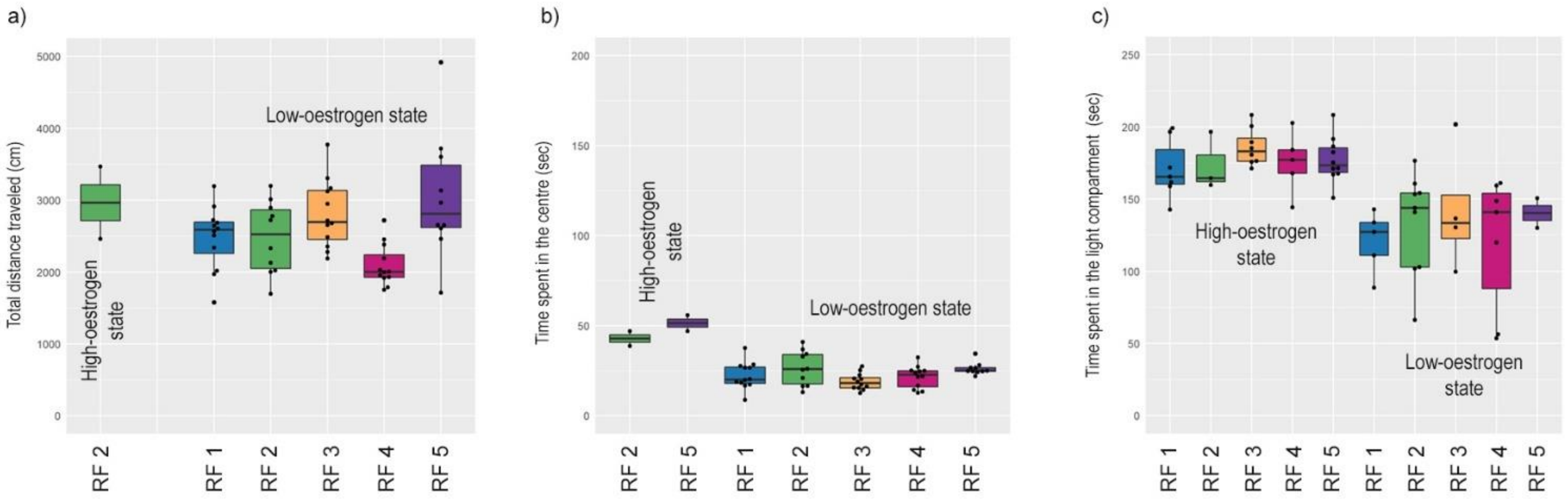

S3 Figure: Oestrous cycle-dependent effects on behavioral phenotype in female mice from different rearing facilities. Results of the open field **(a, b)** and light-dark box **(c)** tests are presented in females depending on the oestrous cycle stage determined immediately after behavioral testing. There was a significant effect of oestrogen status on time spent in the center **(b)** and time spent in the light compartment **(c)** with high-oestrogenic females showing marginally higher activity than low-oestrogenic females **(a)**. The raw data underlying this figure are available in the Figshare repository https://doi.org/10.6084/m9.figshare.21087718.
