# Supplementary material for: The rearing environment persistently modulates mouse phenotypes from the molecular to the behavioural level
Source: PLoS Biol. 2022 Oct 21;20(10):e3001837. doi: 10.1371/journal.pbio.3001837 (PMC9629646; doi:10.1371/journal.pbio.3001837)
Supplement: S4 Fig — Results are presented depending on the oestrous cycle stage determined after HPA reactivity tests. There was a significant effect of oestrogen status on basal corticosterone levels, with high-oestrogenic females showing lower basal corticosterone levels than low estrogenic females. The raw data underlying this figure are available in the Figshare repository https://doi.org/10.6084/m9.figshare.21087799. (PDF) [file pbio.3001837.s016.pdf]

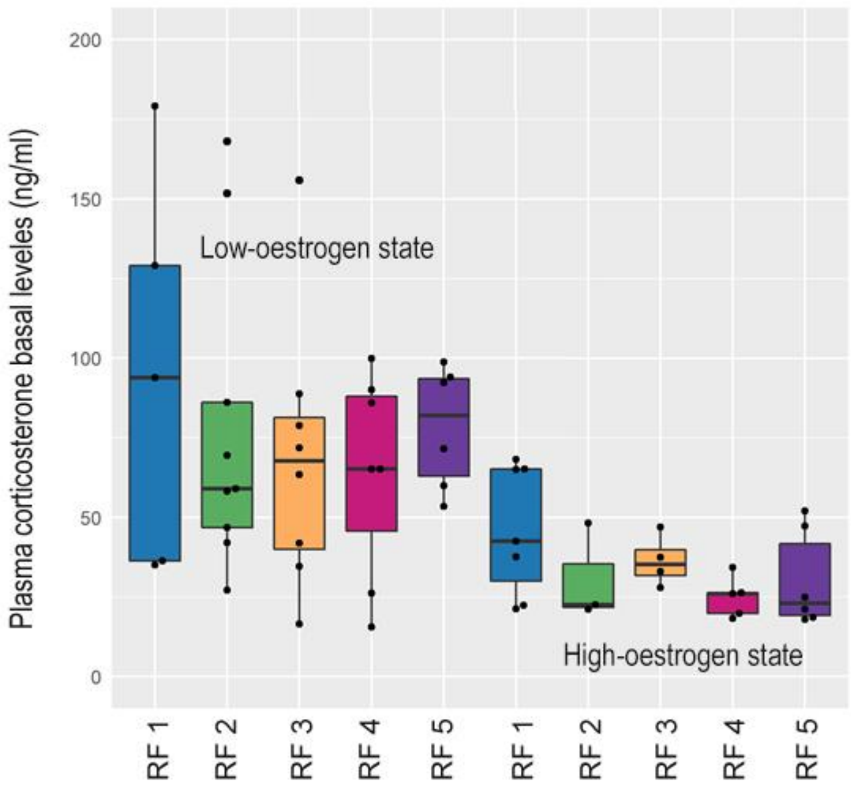

S4 Figure: Oestrous cycle-dependent effects on basal corticosterone levels in female mice from different rearing facilities. Results are presented depending on the oestrous cycle stage determined after HPA reactivity tests. There was a significant effect of oestrogen status on basal corticosterone levels, with high-oestrogenic females showing lower basal corticosterone levels than low estrogenic females. The raw data underlying this figure are available in the Figshare repository https://doi.org/10.6084/m9.figshare.21087799.
