# Supplementary material for: The rearing environment persistently modulates mouse phenotypes from the molecular to the behavioural level
Source: PLoS Biol. 2022 Oct 21;20(10):e3001837. doi: 10.1371/journal.pbio.3001837 (PMC9629646; doi:10.1371/journal.pbio.3001837)
Supplement: S5 Fig — (a) PCoA of the ATAC-seq data. (b) Manhatten distances between samples for closed chromatin sites visualised by t-SNE. (c) The number of significant differentially accessible peaks associated to each of rearing environments in a given TP (one-to-many comparison; adjusted for multiple testing FDR < 0.01 and abs(logFC) > 0.5. (d) The number of significant differentially accessible peaks between groups in a given TP (one-to-one comparison; adjusted for multiple testing FDR < 0.01 and abs(logFC) > 0.5). (e) Chromatin accessibility profiles of the Col19a1, Dlg 2, Fzd9, and Lrrc4c. Shown are genomic coordinates of differential ATAC-seq peaks. The raw data underlying this figure are available from the NCBI GEO database under accession number GSE191125. The analysis script is available at the GitHub repository https://github.com/MWSchmid/Jaric-et-al.-2022. ATAC-seq, assay for transposase-accessible chromatin using sequencing; Dlg 2, discs large homolog 2; FDR, false discovery rate; Fzd9, Frizzled9; GEO, Gene Expression Omnibus; Lrrc4c, Leucine-Rich Repeat-Containing 4C; PCoA, principal coordinate analysis; TP, time point. (PDF) [file pbio.3001837.s017.pdf]

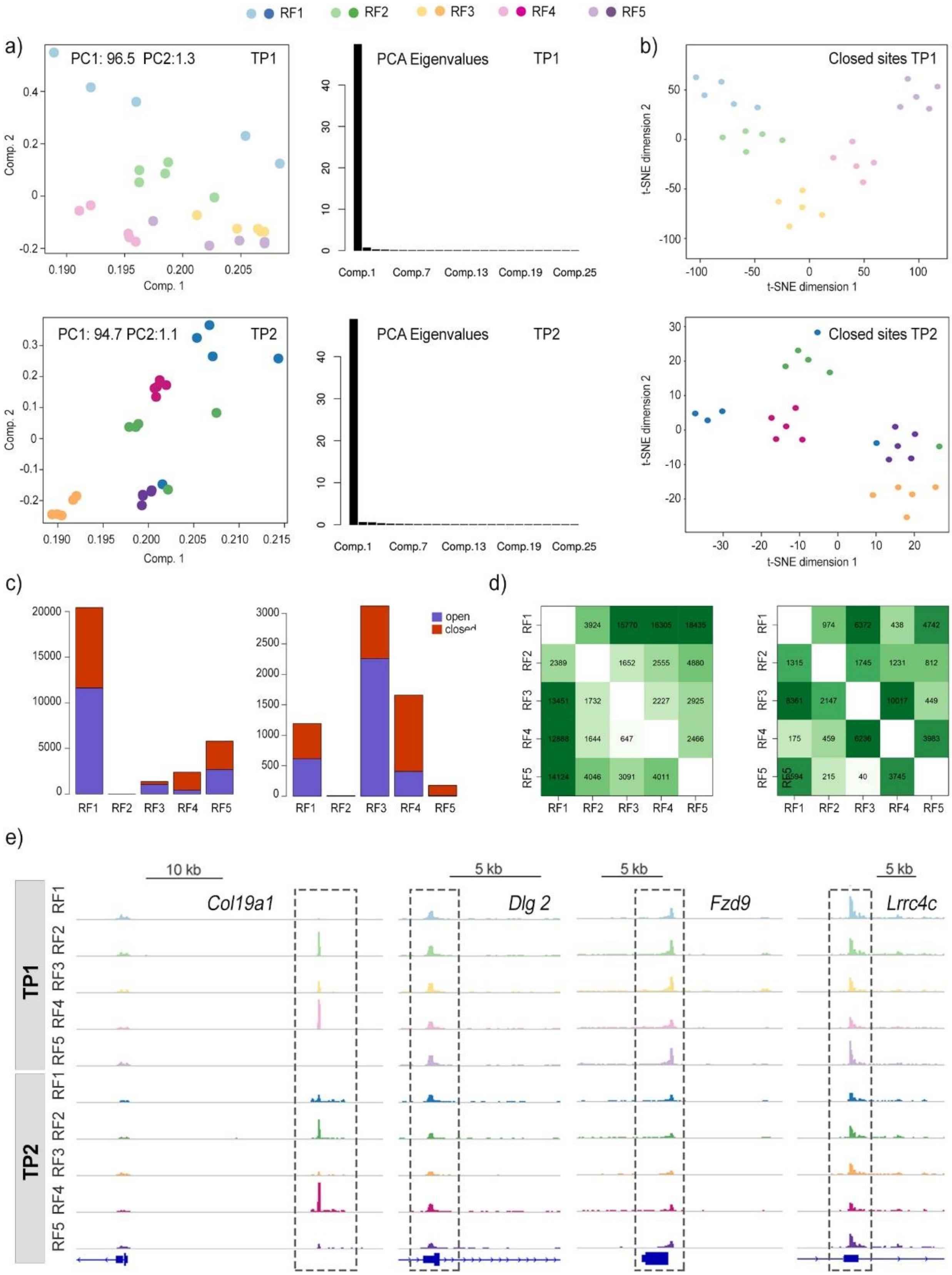

S5 Figure: Neuronal chromatin accessibility differs in males from different rearing facilities. a) Principal component analysis of the ATAC-seq data. **b)** Manhatten distances between samples for closed chromatin sites visualized by t-SNE. **c)** The number of significant differentially accessible peaks associated to each of rearing environments in a given timepoint (one-to-many comparison; adjusted for multiple testing FDR < 0.01 and abs(logFC) > 0.5. **d)** The number of significant differentially accessible peaks between groups in a given timepoint (one-to-one comparison; adjusted for multiple testing FDR < 0.01 and abs(logFC) > 0.5.) **e)** Chromatin accessibility profiles of the *Col19a1*, *Dlg 2*, *Fzd9* and *Lrrc4c*. Shown are genomic coordinates of differential ATAC-seq peaks. The raw data underlying this figure are available from the NCBI Gene Expression Omnibus (GEO) database under accession number GSE191125. The analysis script is available at the GitHub repository https://github.com/MWSchmid/Jaric-et-al.-2022.
