# Supplementary material for: The rearing environment persistently modulates mouse phenotypes from the molecular to the behavioural level
Source: PLoS Biol. 2022 Oct 21;20(10):e3001837. doi: 10.1371/journal.pbio.3001837 (PMC9629646; doi:10.1371/journal.pbio.3001837)
Supplement: S6 Fig — Each pup from a litter is sexed, weighed, and placed in a separate cage/container. After all animals from the litter are checked, it resulted in X number of separate cages/containers with males and Y number of separate cages/containers with females. If the number of males/females in a litter is 1, the animal is not weaned (female in Litter 1); if the number of males/females is 2 or 3, they are all weaned into same sex groups and taken to the housing room (Litter 2 and males in Litter 3); and if the number of males/females is >3, the 3 animals to be housed together are chosen using a random number generator (males in Litter 1 and females in Litter 3). Unweaned pups (female in Litter 1), extra pups, and dams were killed after the pups have been weaned. (PDF) [file pbio.3001837.s018.pdf]

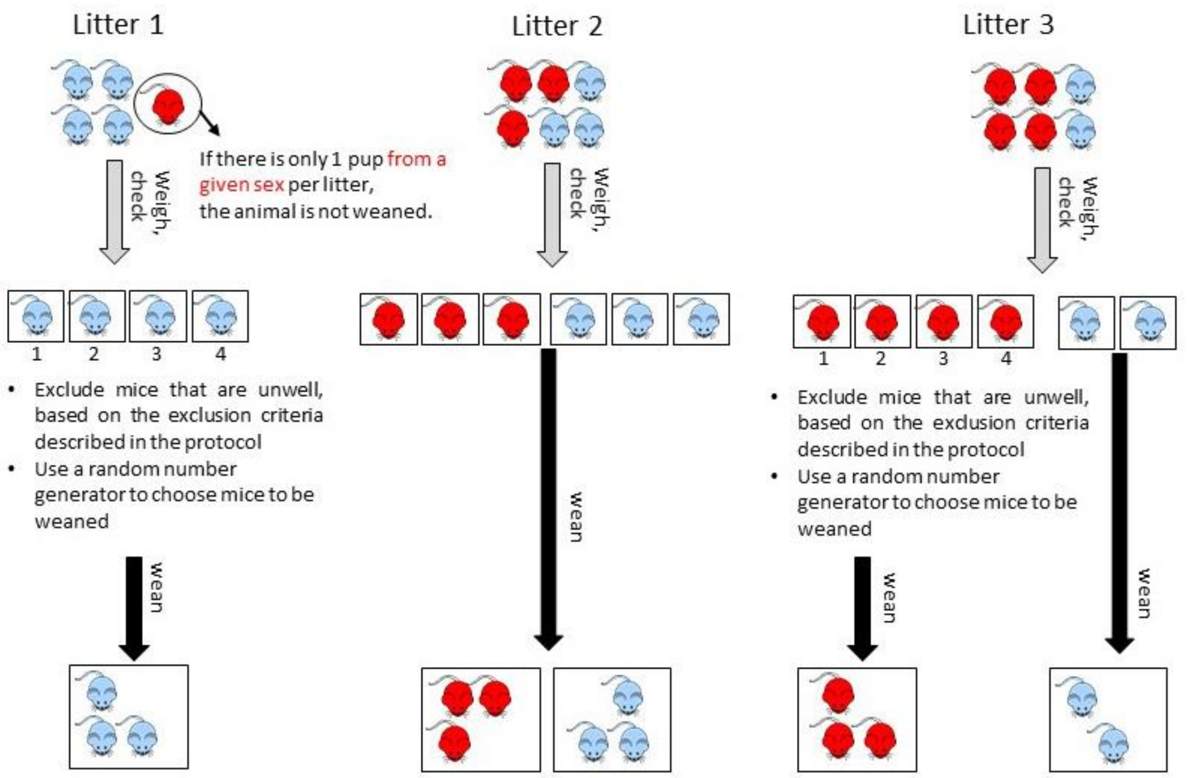

The weaning criteria to form groups of same-sex littermates:

1. **All litters with 3 or more males are used to wean 3 male littermates.**
2. **All litters with 3 or more females are used to wean 3 female littermates.**
3. **Additionally, any litters with 2 males or 2 females should also be weaned into same sex groups.**

S6 Figure: Weaning strategy. The each pup from a litter is sexed, weighed and placed in a separate cage/container. After all animals from the litter are checked, it resulted in X number of separate cages/containers with males and Y number of separate cages/containers with females.
If the number of males/females in a litter is 1, the animal is not weaned (female in Litter 1);
If the number of males/females is 2 or 3, they are all weaned into same sex groups and taken to the housing room (Litter 2 and males in Litter 3);
If the number of males/females is > 3, the three animals to be housed together are chosen using a random number generator (males in Litter 1 and females in Litter 3);
Unweaned pups (female in Litter 1), extra pups and dams were euthanized after the pups have been weaned.
