# Supplementary material for: The rearing environment persistently modulates mouse phenotypes from the molecular to the behavioural level
Source: PLoS Biol. 2022 Oct 21;20(10):e3001837. doi: 10.1371/journal.pbio.3001837 (PMC9629646; doi:10.1371/journal.pbio.3001837)
Supplement: S7 Fig — (a) Histogram of individual library sizes. (b) Example rarefaction plots of 8 randomly selected samples. The red line indicates rarefaction depth. The raw data underlying this figure are available in the Figshare repository https://doi.org/10.6084/m9.figshare.21087877. The 16S rRNA gene sequencing data are available from the European Nucleotide Archive (ENA) under accession number PRJEB49361. (PDF) [file pbio.3001837.s019.pdf]

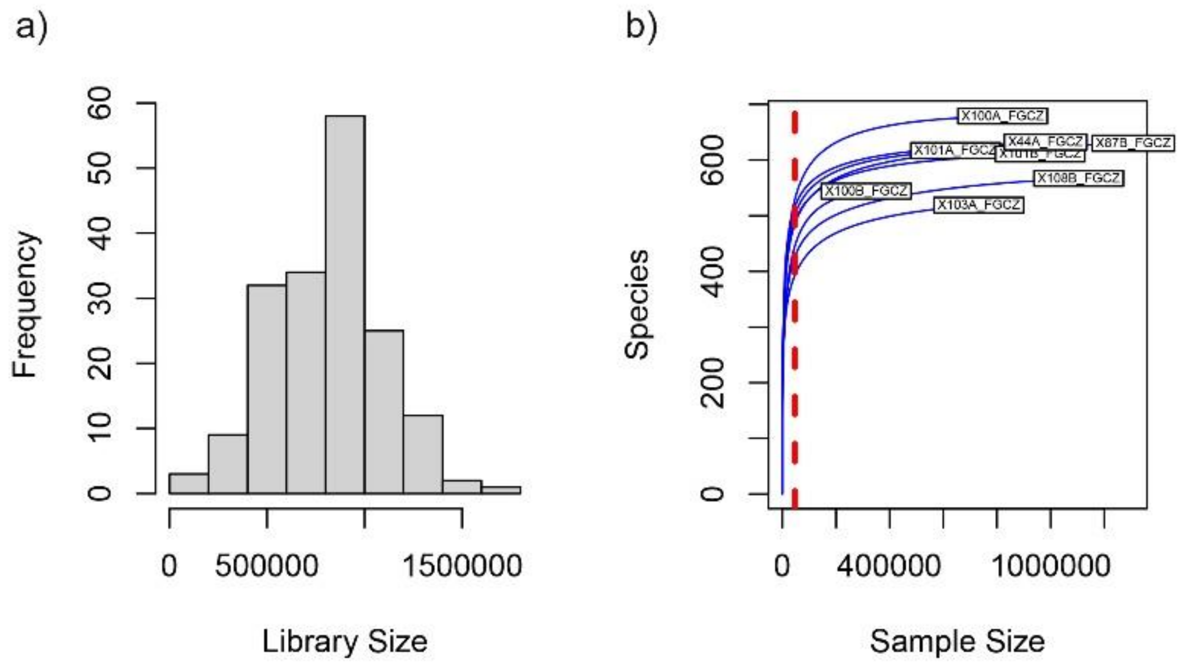

S7 Figure: Random down-sampling of 16S sequencing reads. a) Histogram of individual library sizes. **b)** Example rarefaction plots of 8 randomly selected samples. The red line indicates rarefaction depth. The raw data underlying this figure are available in the Figshare repository https://doi.org/10.6084/m9.figshare.21087877. The 16S rRNA gene sequencing data are available from the European Nucleotide Archive (ENA) under accession number PRJEB49361.
