# Supplementary material for: The rearing environment persistently modulates mouse phenotypes from the molecular to the behavioural level
Source: PLoS Biol. 2022 Oct 21;20(10):e3001837. doi: 10.1371/journal.pbio.3001837 (PMC9629646; doi:10.1371/journal.pbio.3001837)
Supplement: S8 Fig — (a) Mean values for α-diversity metrics for MA mice and BT mice. Top left: Chao1 richness; bottom left: observed species richness; top right: Pielou evenness, bottom right: Shannon diversity index. (b) Results of Wilcoxon signed-rank test for each α-diversity metric between MA and BT mice. (c) Ordination plot visualizing PCoA based on Bray–Curtis dissimilarity between samples from MA and BT mice collected at TP2. (d) Result of PERMANOVA partitioning variation in microbiome composition between mice used for MA and BT. The raw data underlying this figure are available in the Figshare repository https://doi.org/10.6084/m9.figshare.21087931. The 16S rRNA gene sequencing data are available from the ENA under accession number PRJEB49361. ENA, European Nucleotide Archive; PCoA, principal coordinate analysis; PERMANOVA, permutational analysis of variance. (PDF) [file pbio.3001837.s020.pdf]

a)

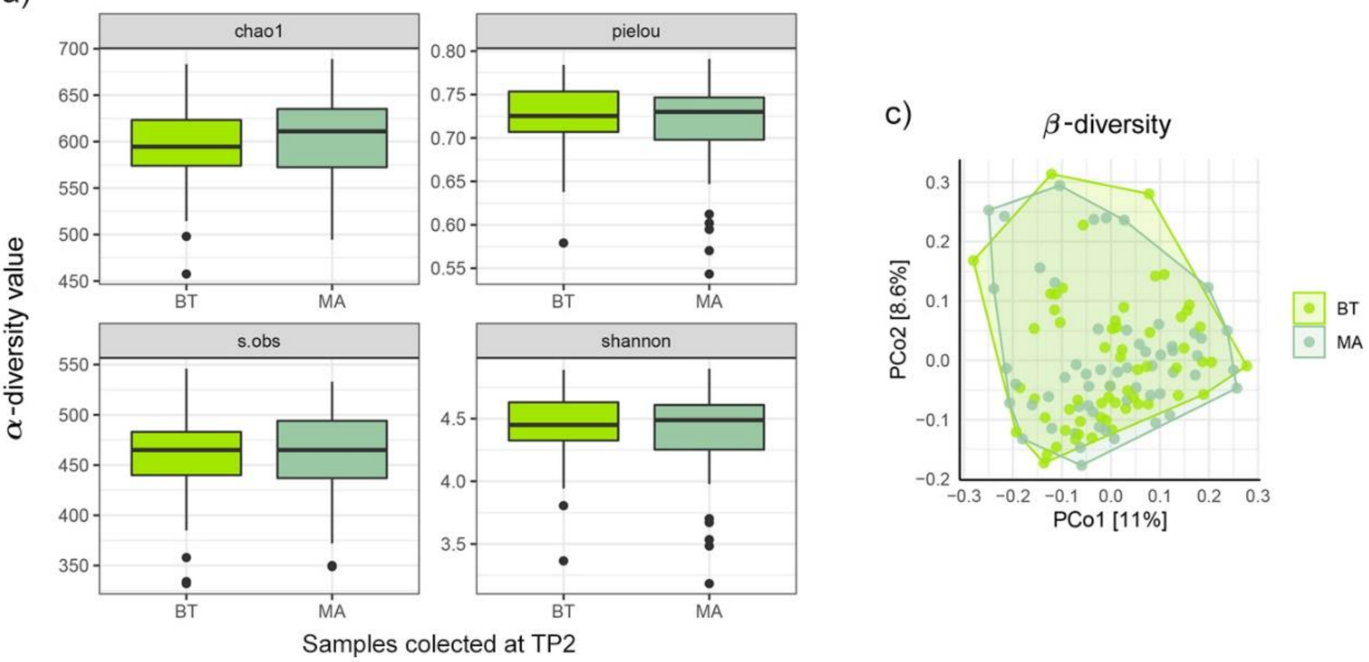

b)

Diversity metric	test statistics	p value
Chao 1 richness	1542	0.4427
Pielou evenness	1738	0.7571
Observed richness	1601	0.6586
Shannon diversity	1736	0.7655

d)

	Df	SumofSqs	R2	F	Pr(>F)
Animals for MA or BA	1	0.06047	0.003793	0.434	1
Residuals	114	15.88	0.9962	NA	NA
Total	115	15.94	1	NA	NA

S8 Figure: Evaluation of differences in microbiome between samples from behaviorally tested-BT mice and mice used for chromatin profiling-MA mice. a) Mean values for α-diversity metrics for MA-mice and BT-mice. Top left-Chao1 richness, bottom left-Observed species richness, top right-Pielou evenness, bottom right-Shannon diversity index. **b)** Results of Wilcoxon signed-rank test for each α-diversity metric between MA and BT mice. **c)** Ordination plot visualizing Principal Coordinate analysis based on Bray-Curtis dissimilarity between samples from MA and BT-mice collected at TP2. **d)** Result of PERMANOVA partitioning variation in microbiome composition between mice used for MA and BT.

The raw data underlying this figure are available in the Figshare repository https://doi.org/10.6084/m9.figshare.21087931. The 16S rRNA gene sequencing data are available from the European Nucleotide Archive (ENA) under accession number PRJEB49361.

.
