# Supplementary material for: The rearing environment persistently modulates mouse phenotypes from the molecular to the behavioural level
Source: PLoS Biol. 2022 Oct 21;20(10):e3001837. doi: 10.1371/journal.pbio.3001837 (PMC9629646; doi:10.1371/journal.pbio.3001837)
Supplement: S9 Fig — Power curve for a one-way ANOVA with α = 0.05, k = 5 rearing facilities and n = 6 to 30 subjects, assuming an average means difference between 2 randomly chosen labs of 10% (blue), 20% (orange), or 30% (green). Power estimates are based on 10,000 repeated samples. To generate differences between labs we sampled distribution means from a normal distribution with the reported mean (10,000 square units) and standard deviation of 890, 1,780, and 2,260 square units, which resulted in samples, where the difference in the effect size between 2 randomly chosen labs was on average 10%, 20%, or 30%. The numerical data and code underlying this figure are available in the Figshare repository https://doi.org/10.6084/m9.figshare.21087931. (PDF) [file pbio.3001837.s021.pdf]

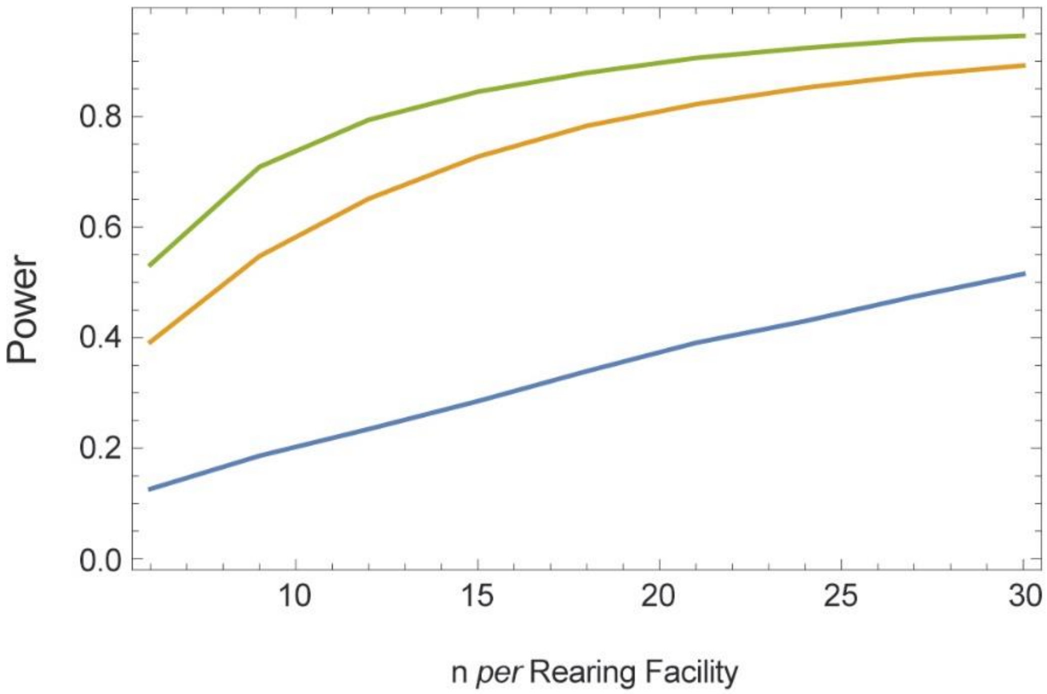

S9 Figure: Power analysis: Power curve for a one-way ANOVA with α=0.05, k=5 rearing facilities and n=6 to 30 subjects, assuming an average means difference between two randomly chosen labs of 10% (blue), 20% (orange) or 30% (green). Power estimates are based on 10, 000 repeated samples. To generate differences between labs we sampled distribution means from a normal distribution with the reported mean (10,000 square units) and standard deviation of 890, 1780, and 2260 square units, which resulted in samples, where the difference in the effect size between two randomly chosen labs was on average 10, 20, or 30 percent. The numerical data and code underlying this figure are available in the Figshare repository https://doi.org/10.6084/m9.figshare.21087931.
