# Supplementary material for: The rearing environment persistently modulates mouse phenotypes from the molecular to the behavioural level
Source: PLoS Biol. 2022 Oct 21;20(10):e3001837. doi: 10.1371/journal.pbio.3001837 (PMC9629646; doi:10.1371/journal.pbio.3001837)
Supplement: S10 Fig — The data points of the body weights were normally distributed both in males (top panel) and females (bottom panel). The underlying numerical data are available in Fig 2 Data (Fig 2A MALES; Fig 2A FEMALES) in the Figshare repository https://doi.org/10.6084/m9.figshare.21081949. (PDF) [file pbio.3001837.s022.pdf]

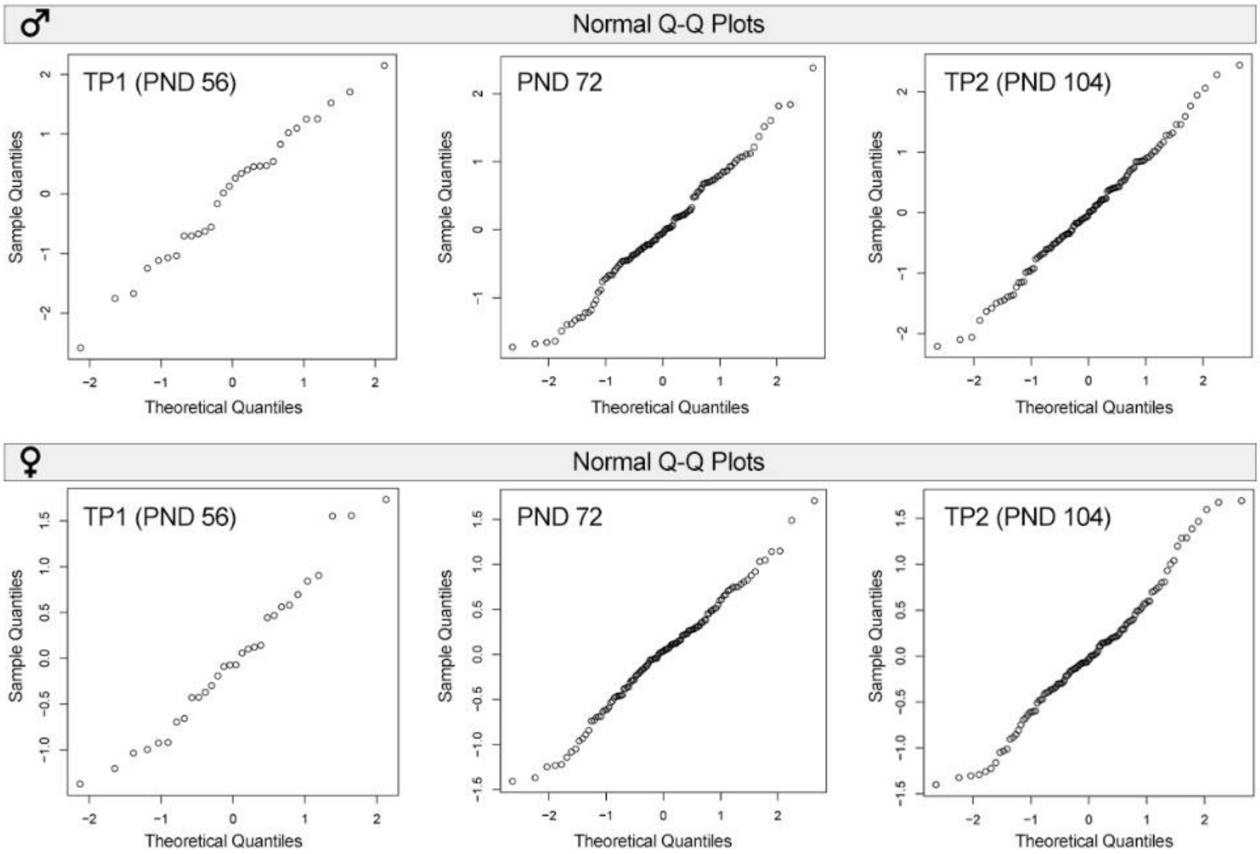


**S10 Figure: Q-Q (quantile-quantile) a probability plots for the body weight data points.** The data points of the body weights were normally distributed both in males (top panel) and females (bottom panel). The underlying numerical data are available in Figure 2 Data (Figure 2a MALES; Figure 2a FEMALES) in the Figshare repository https://doi.org/10.6084/m9.figshare.21081949.
