# Supplementary material for: The rearing environment persistently modulates mouse phenotypes from the molecular to the behavioural level
Source: PLoS Biol. 2022 Oct 21;20(10):e3001837. doi: 10.1371/journal.pbio.3001837 (PMC9629646; doi:10.1371/journal.pbio.3001837)
Supplement: S11 Fig — The data points of the corticosterone response were normally distributed both in males (top panel) and females (bottom panel). The underlying numerical data are available in Fig 2 Data (Fig 2C MALES; Fig 2C FEMALES) in the Figshare repository https://doi.org/10.6084/m9.figshare.21081949. (PDF) [file pbio.3001837.s023.pdf]

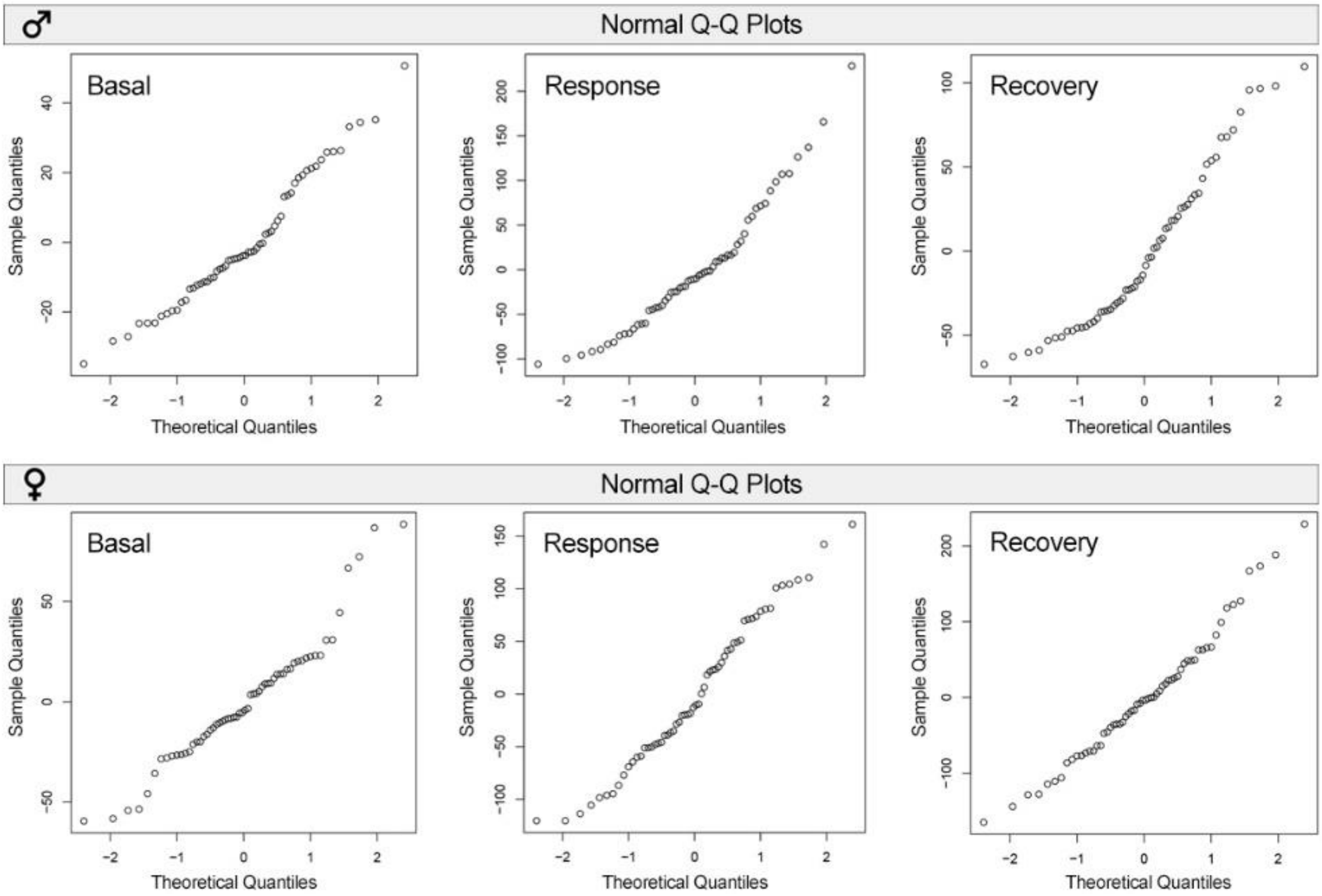

S11 Figure: Q-Q (quantile-quantile) a probability plots for the corticosterone response data points in the stress reactivity tests. The data points of the corticosterone response were normally distributed both in males (top panel) and females (bottom panel). The underlying numerical data are available in Figure 2 Data (Figure 2c MALES; Figure 2c FEMALES) in the Figshare repository https://doi.org/10.6084/m9.figshare.21081949.
