# Supplementary material for: The rearing environment persistently modulates mouse phenotypes from the molecular to the behavioural level
Source: PLoS Biol. 2022 Oct 21;20(10):e3001837. doi: 10.1371/journal.pbio.3001837 (PMC9629646; doi:10.1371/journal.pbio.3001837)
Supplement: S12 Fig — The data points of the relative adrenal gland weights were normally distributed both in males (left plot) and females (right plot). The underlying numerical data are available in Fig 2 Data (Fig 2D MALES; Fig 2D FEMALES) in the Figshare repository https://doi.org/10.6084/m9.figshare.21081949. (PDF) [file pbio.3001837.s024.pdf]

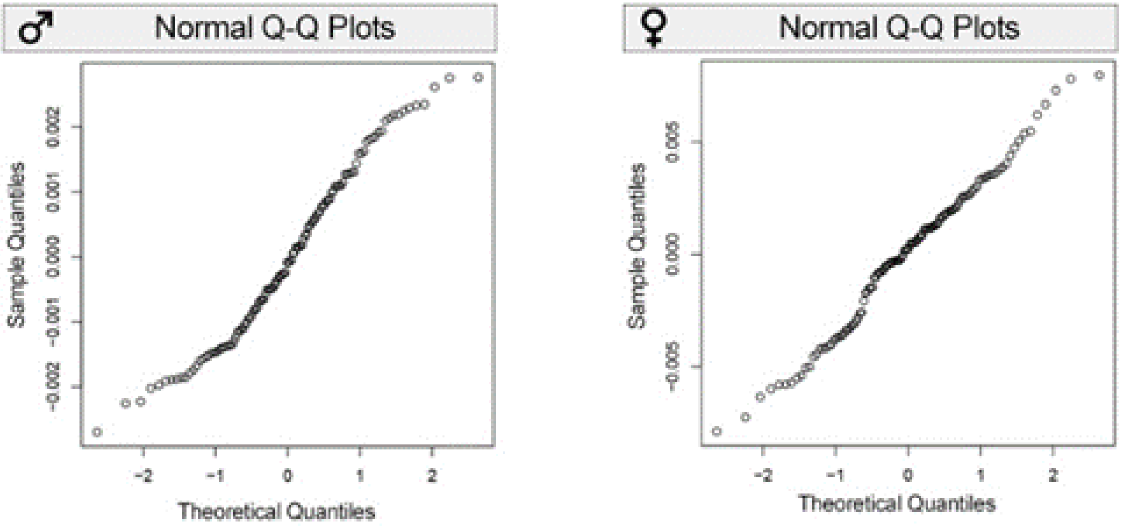

S12 Figure: Q-Q (quantile-quantile) a probability plots for the relative adrenal gland weight at TP2. The data points of the relative adrenal gland weights were normally distributed both in males (left plot) and females (right plot). The underlying numerical data are available in Figure 2 Data (Figure 2d MALES; Figure 2d FEMALES) in the Figshare repository https://doi.org/10.6084/m9.figshare.21081949.
