# Supplementary material for: The rearing environment persistently modulates mouse phenotypes from the molecular to the behavioural level
Source: PLoS Biol. 2022 Oct 21;20(10):e3001837. doi: 10.1371/journal.pbio.3001837 (PMC9629646; doi:10.1371/journal.pbio.3001837)
Supplement: S13 Fig — Q–Q plots are presented before and after transformation of individual data points for both males (a) and (b) females. The underlying numerical data are available in Fig 2 Data (Fig 2B MALES; Fig 2B FEMALES) in the Figshare repository https://doi.org/10.6084/m9.figshare.21081949. (PDF) [file pbio.3001837.s025.pdf]

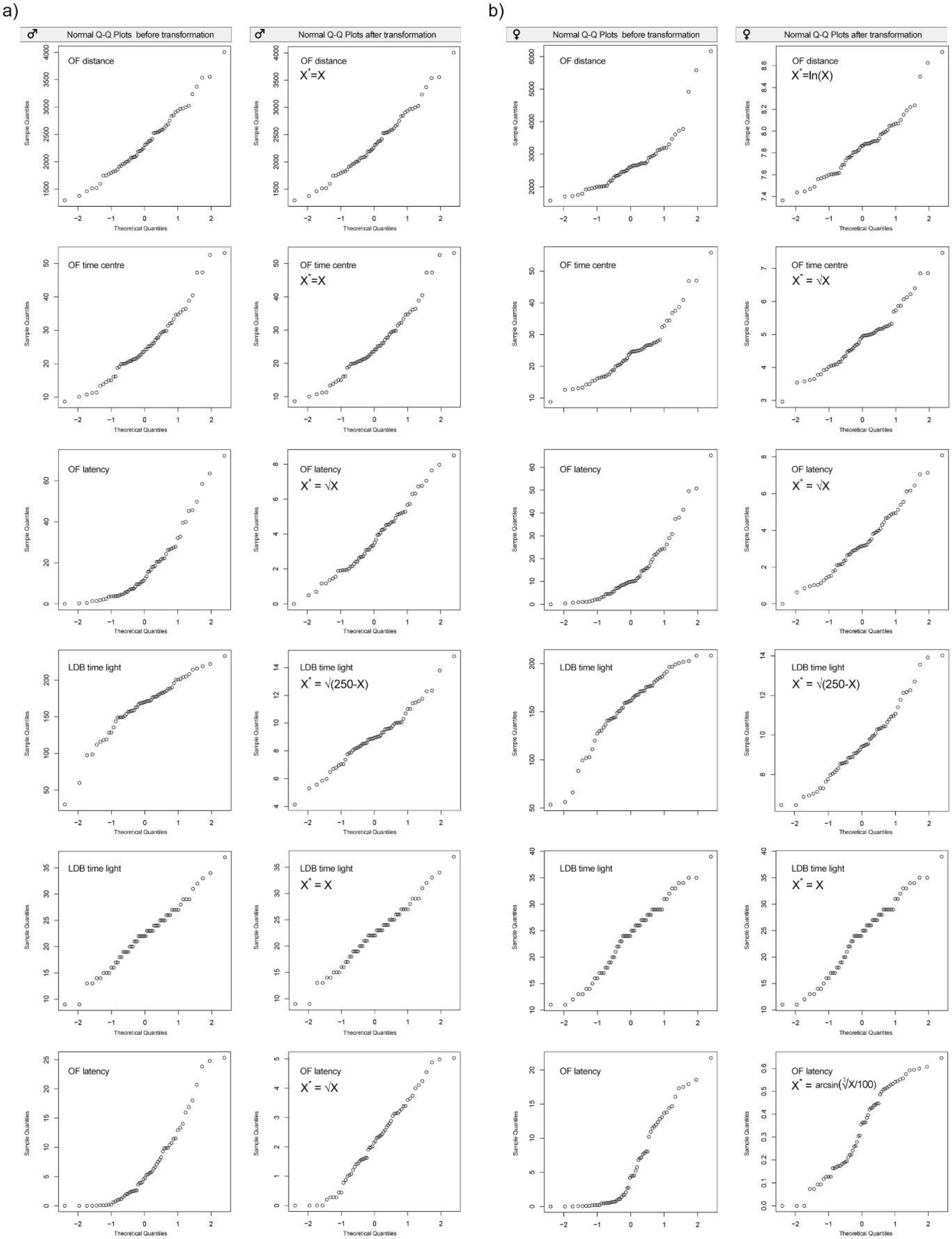

S13 Figure: Q-Q (quantile-quantile) a probability plots for the behavioral data sets. Q-Q plots are presented before and after transformation of individual data points for both males **(a)** and **(b)** females. The underlying numerical data are available in Figure 2 Data (Figure 2b MALES; Figure 2b FEMALES) in the Figshare repository https://doi.org/10.6084/m9.figshare.21081949.
