# Supplementary material for: The rearing environment persistently modulates mouse phenotypes from the molecular to the behavioural level
Source: PLoS Biol. 2022 Oct 21;20(10):e3001837. doi: 10.1371/journal.pbio.3001837 (PMC9629646; doi:10.1371/journal.pbio.3001837)
Supplement: S15 Fig — Sorting plots from 3 negative controls (nuclei only, DAPI only, and Isotype control + DAPI) processed without primary antibody (neuron-specific marker NeuN), positive control containing NeuN antibody conjugated with Alexa 488 only (NeuN-Alexa 488 only control) and our sample processed with NeuN-Alexa 488 antibody and DAPI are shown. Representative FANS reports showing the gating strategy for the checking the size and granularity, removal of debris, and ensuring a successful separation of NeuN+ (neuronal) from nonneuronal single nuclei. (PDF) [file pbio.3001837.s027.pdf]

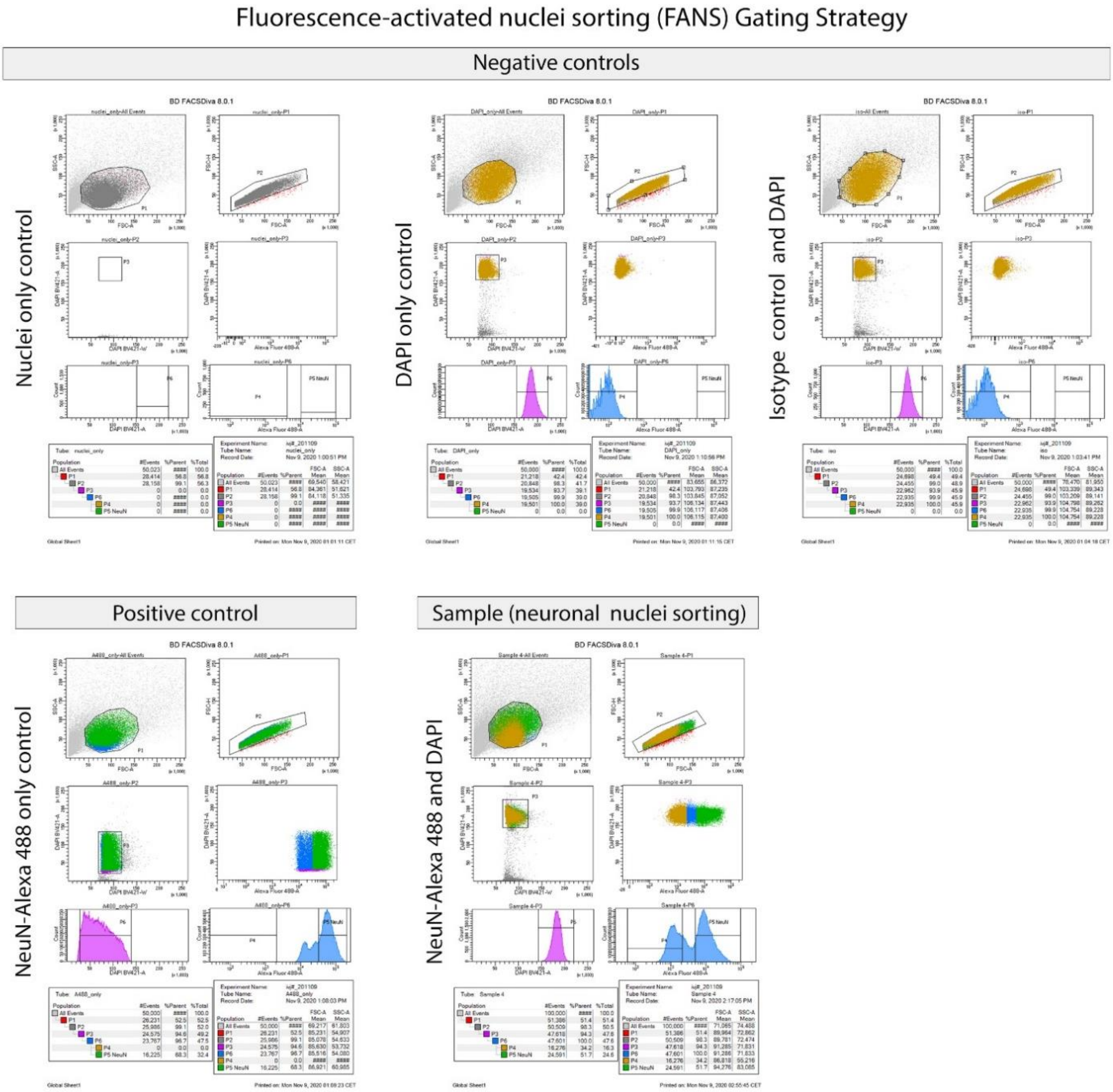

S15 Figure: Gating strategy for separation of neuronal nuclei using fluorescence-activated nuclei sorting (FANS). Sorting plots from **three negative controls** (Nuclei only, DAPI only and Isotype control + DAPI) processed without primary antibody (neuron-specific marker NeuN), **positive control** containing NeuN antibody conjugated with Alexa 488 only (NeuN-Alexa 488 only control) and our **sample** processed with NeuN-Alexa 488 antibody and DAPI are shown. Representative FANS reports showing the gating strategy for the checking the size and granularity, removal of debris and ensuring a successful separation of NeuN+ (neuronal) from non-neuronal single nuclei.
