# Supplementary material for: The rearing environment persistently modulates mouse phenotypes from the molecular to the behavioural level
Source: PLoS Biol. 2022 Oct 21;20(10):e3001837. doi: 10.1371/journal.pbio.3001837 (PMC9629646; doi:10.1371/journal.pbio.3001837)
Supplement: S16 Fig — The quality control of ATAC-seq libraries was performed by using Fragment Analyzer (FA). The representative FA trace with nucleosomal banding pattern is shown. (PDF) [file pbio.3001837.s028.pdf]

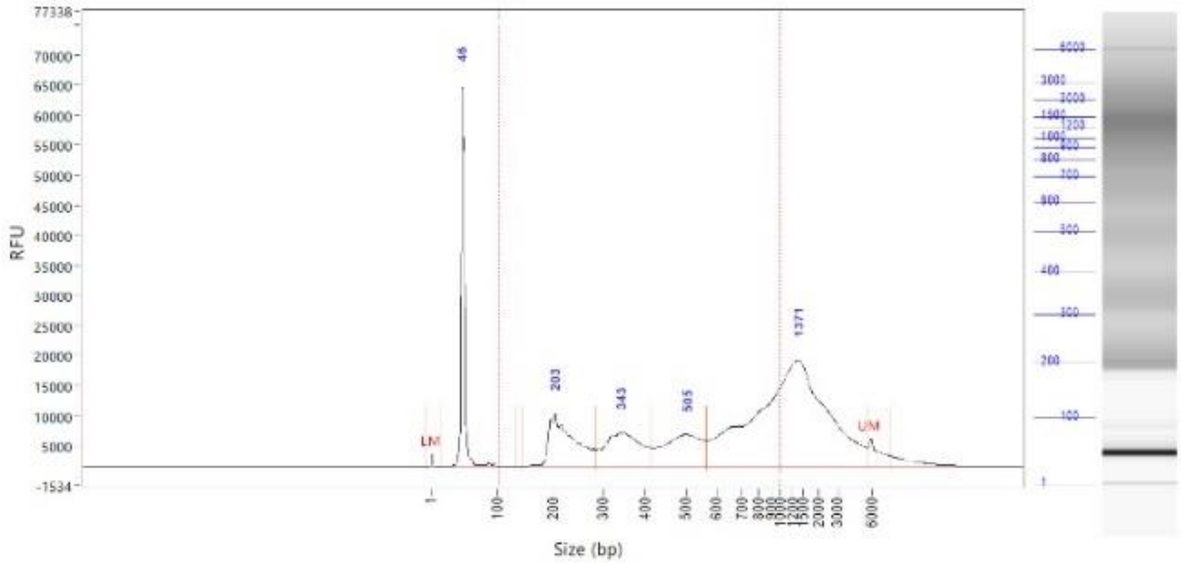

**S16 Figure: ATAC-seq library quality control.** The quality control of ATAC-seq libraries was performed by using Fragment Analyzer (FA). The representative FA trace with nucleosomal banding pattern is shown.
