# Supplementary material for: The rearing environment persistently modulates mouse phenotypes from the molecular to the behavioural level
Source: PLoS Biol. 2022 Oct 21;20(10):e3001837. doi: 10.1371/journal.pbio.3001837 (PMC9629646; doi:10.1371/journal.pbio.3001837)
Supplement: S17 Fig — The number of peaks drops quite strongly with the minimal number of samples required for merging peaks. (a) Representative scatter plots showing log2 (x + 1) transformed, normalised values averaged in RF1 and RF2 at TP1. (b) Only high-confidence broad peaks, shared across at least 3 biological replicates of 1 group are used for all downstream analyses. The raw data underlying this figure are available from the NCBI Gene Expression Omnibus (GEO) database under accession number GSE191125. The analysis script is available at the GitHub repository https://github.com/MWSchmid/Jaric-et-al.-2022. (PDF) [file pbio.3001837.s029.pdf]

a)

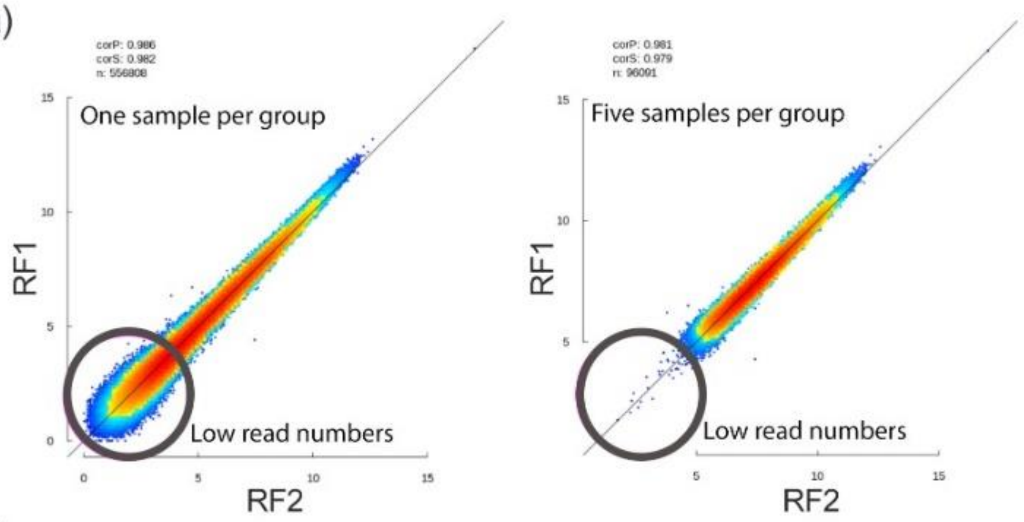

b)

TP1			TP 2		
Rearing Laboratory	Peak in at least X samples	Number of Peaks	Rearing Laboratory	Peak in at least X samples	Number of Peaks
RF1	1	198999	RF1	1	265210
	2	137897		2	117035
	**3**	**113319**		**3**	**80246**
	4	95434		4	58879
	5	77201		5	33177
RF2	1	215969	RF2	1	275804
	2	146670		2	120220
	**3**	**119601**		**3**	**82659**
	4	100111		4	61167
	5	81027		5	38839
RF3	1	188900	RF3	1	292225
	2	119987		2	132363
	**3**	**96666**		**3**	**93581**
	4	81002		4	72337
	5	65749		5	53763
RF4	1	207986	RF4	1	251995
	2	121719		2	117417
	**3**	**94068**		**3**	**82628**
	4	76680		4	63738
	5	60496		5	47639
RF5	1	197735	RF5	1	259394
	2	124138		2	140724
	**3**	**98970**		**3**	**106509**
	4	81939		4	86395
	5	65515		5	68095

S17 Figure: ATAC peak count statistics. The number of peaks drops quite strongly with the minimal number of samples required for merging peaks. a) Representative scatter plots showing log2 (x+1) transformed, normalized values averaged in RF1 and RF2 at TP1. b) Only high-confidence broad peaks, shared across at least **three biological replicates of one group** are used **for all** downstream analyses. The raw data underlying this figure are available from the NCBI Gene Expression Omnibus (GEO) database under accession number GSE191125. The analysis script is available at the GitHub repository https://github.com/MWSchmid/Jaric-et-al.-2022.
