# Supplementary material for: The rearing environment persistently modulates mouse phenotypes from the molecular to the behavioural level
Source: PLoS Biol. 2022 Oct 21;20(10):e3001837. doi: 10.1371/journal.pbio.3001837 (PMC9629646; doi:10.1371/journal.pbio.3001837)
Supplement: S1 Text — (PDF) [file pbio.3001837.s034.pdf]

Supporting text

The rearing environment persistently modulates mouse phenotypes from the molecular to the behavioural level

Ivana Jaric, Bernhard Voelkl, Melanie Clerc, Marc W. Schmid, Janja Novak, Marianna Rosso1, Reto Rufener, Vanessa Tabea von Kortzfleisch, S. Helene Richter, Manuela Buettner, André Bleich, Irmgard Amrein, David P. Wolfer, Chadi Touma, Shinichi Sunagawa, Hanno Würbel

Sample size: For the confirmatory part of the study (behavioural and physiological outcomes) appropriate sample sizes were determined a priori in a power analysis using simulated sampling for a two-way ANOVA design. The power analysis was done for the main outcome variable, plasma corticosterone levels in the SRT. Based on historical data (1,2), we expected to observe an effect of medium size (i.e. means estimates for two randomly chosen rearing facilities are expected to be in the range of 20%, equivalent to a ratio of between-facility: within-facility variation of 1:2). This resulted in a required minimal sample size of 12 mice per rearing facility and sex (n=120).

For the exploratory part of the study (gut microbiota and ATAC seq analyses) no statistical methods were used to predetermine sample size. For gut microbiota analysis, samples were taken at both time points (TPs), at each rearing facility (RF) and at the end of the experiment at the testing laboratory. Six cages per sex and rearing facility were randomly selected from all cages contained 3 littermates after weaning (n=180). One mouse from each selected cage was sacrificed in the rearing facility at 8 weeks of age (PND 56; TP1; n=60), while the other two cage mates (n=120; one underwent behavioral testing and one remained naïve) were sacrificed in the testing laboratory at TP2 (14.5 weeks of age; PND 102).

The ATAC-seq analysis was performed at both time points. For this, test-naïve male mice were used to avoid effects of testing on the chromatin profile. Five cages per rearing facility were selected randomly from all cages containing 3 male littermates after weaning. One mouse from the selected cages was sacrificed in the rearing facility at TP1 (total n=25, i.e. 5 biological replicates per rearing laboratory), while its test-naïve littermate was sacrificed in the testing laboratory at the end of the experiment (TP2; total n=25, i.e. 5 biological replicates per rearing facility).

Data exclusion: No data were excluded. The squared Mahalanobis distance analysis suggested the existence of 6 outliers in the male cohort and 6 in the female cohort; S9 Figure), which were not removed for the further analysis. The outliers had no impact on the results which was confirmed by re-analysis with the outliers removed.

Replication: We used biological replicates in all experiments.

Randomization: The order of the cages during animal habituation and testing of all mice was randomized using the random number generator of the software Mathematica (version 11). Each of the 3 experimenters got randomly assigned 20 male and 20 female mice which they handled during the behavioural testing. Always two experimenters were testing animals in parallel (at the same time, but in separate apparatuses) by two experimenters, with 3 different combinations of 2 experimenters each day. Testing was carried out in batches during four consecutive days. The randomization and allocation procedures were restricted so that in each block for each experimenter there is exactly one from each rearing facility in random order, with the addition that in no case animals tested at the same time are from the same rearing facility. The allocation and randomization script are available as Supporting file “R-markdown and Mathematica code”.

Blinding: All experimenters performing the stress reactivity tests, behavioural tests, and post-mortem analyses were be blind to the "treatment", i.e. the rearing facility the animals were transferred from. Blinding was done by two colleagues otherwise not involved in the execution

of the experiments. Cages were assigned new identification numbers and positions of cages within and between the cage racks were randomly re-shuffled so that the experimenters cannot deduce the origin of the cages (i.e. rearing facility) from the ID number or the position of the cage. For assignment of ID numbers and cage positions a script was written in the software Mathematica (version 11), using the inbuilt random number generator.

ATAC Seq Data deposition: The raw data underlying this figure are available from the NCBI Gene Expression Omnibus (GEO) database under accession number GSE191125. The analysis script is available at the GitHub repository https://github.com/MWSchmid/Jaric-et-al.-2022

Files in database submission: Unprocessed read files in FASTQ format (forward and reverse, one pair per sample), called peaks in BED format (one per sample), merged peaks in BED format, counts per peak and sample in CSV format and normalized counts per peak and sample in CSV format.

Sequencing depth: Paired-end reads (2*100 bp), 3.28 billion read pairs in total, 65.7 million pairs per sample on average, unique alignment rate on average 80 %.

Peak calling parameters: Sequencing reads were trimmed, and quality checked with fastp (version 0.20.1) with CTGTCTCTTATACACATCT as adapter sequence and a minimal read length of 30 bp. Reads were aligned to the mouse reference genome (ensembl build 102) with Bowtie2 in paired end mode (version 2.3.5.1) keeping only concordant and unique alignments. Duplicate read pairs were marked using the MarkDuplicates command from the Picard software suite (version 1.140). Peaks were then called in each sample separately with MACS2 (version 2.1.4, with the parameters -f BAMPE -g mm --nomodel -q 0.05 --broad --broad-cutoff 0.1 --keep-dup all) as previously reported (github.com/macs3-project/MACS/issues/145). For each time point and rearing facility, peaks were intersected with multovl (version 1.3; and only peaks found in at least three samples per group (i.e. rearing laboratory) were kept. Finally, peaks from all groups were merged with multovl (union of all peaks within groups). The number of reads within peak intervals was obtained with featureCounts (version 2.0.1) with the parameters --primary --ignoreDup --minOverlap 30.

Data quality: All individual peaks had FDR < 0.05. Only peaks found in at least three out of five replicates were used for downstream analysis. Variation in read counts was analyzed with a general linear model in R (version 3.6.1) with the package DESeq2 (version 1.24.0) according to a factorial design with the two explanatory factors "rearing facility (RF)" and "processing batch", within each timepoint. For the annotation of peaks, we used the ChiPseeker annotation for the plot with genomic features and the Homer annotation for TSS distance and candidate, protein coding, genes. Following specific conditions were compared with linear contrasts: i) one-to-one (oto) comparison of each pair of laboratories (RF1 vs RF2, RF1 vs RF3, etc) for each time point; ii) one-to-many (otm) comparison of one laboratory to all other laboratories for each time point (RF1 vs all other RFs, RF2 vs all other RFs, etc), and iii) a global test for the factor "rearing facility" (LRT_RF), i.e. do different rearing laboratories differ in general. Within each comparison, p-values were adjusted for multiple testing (Benjamini-Hochberg), and regions with an adjusted p-value (false discovery rate, FDR) below 0.01 and a minimal log2 fold-change (i.e. the difference between the log2 transformed, normalized sequence counts) of 0.5 were considered to be differentially accessible.

1. Herman JP, Cullinan WE, Morano MI, Akil H, Watson SJ. Contribution of the ventral subiculum to inhibitory regulation of the hypothalamo-pituitary-adrenocortical axis. J Neuroendocrinol. 1995 Jun;7(6):475–82.
2. Fediuc S, Campbell JE, Riddell MC. Effect of voluntary wheel running on circadian corticosterone release and on HPA axis responsiveness to restraint stress in Sprague-Dawley rats. J Appl Physiol (1985). 2006 Jun;100(6):1867–75.
