# Supplementary material for: The rearing environment persistently modulates mouse phenotypes from the molecular to the behavioural level
Source: PLoS Biol. 2022 Oct 21;20(10):e3001837. doi: 10.1371/journal.pbio.3001837 (PMC9629646; doi:10.1371/journal.pbio.3001837)
Supplement: Attachment — Submitted filename: Response to Reviewers.docx [file pbio.3001837.s035.docx]

**Response to Reviewer’s Comments PBIOLOGY-D-22-01111R1**

We would like to thank the reviewers for their time and effort as well as for thoughtful comments and suggestions, which were helpful in improving the manuscript. As suggested by the editor, we have changed the title and the last sentence of the abstract. We also provide additional discussion of the points raised by reviewer 3 and addressed inquiries by reviewer 1. Our revised manuscript includes one new supporting table (S 11 Table), one additional supporting figure (S6 Figure), and about 330 words of added text with changes highlighted.

Below please find a point-by-point response to all reviewers' comments. Thank you very much for your continued consideration of our manuscript.

Ivana Jaric and Hanno Würbel (on behalf of all the authors)

**Reviewer #1:**

In the present study, the investigators utilized an innovative systematic multi-center approach to evaluate the effects of rearing environment on several behavioral and physiological endpoints in C57BL/6J mice. Briefly, the authors allocated and shipped pregnant mice from the same breeding stock to five independent animal facilities where their offspring were reared until eight weeks of age. After this period, all mice were transferred to a sixth and independent laboratory where they were evaluated on multiple endpoints of interest. This study design allowed the investigators to separate the impact of genetics and other environmental factors that can impact study outcomes at the time of testing to obtain a clearer understanding of the role of the rearing environment. There were many endpoints affected by rearing environment, for example a persistence in microbiome diversity differences, in addition to differences in body weights and behavioral phenotypes. Overall, this is a very nice study that provides some important information about variation between rearing environments and the limitations of studies originating from single laboratories. I only have a few comments/considerations listed below:

**1.** The authors did a great job with their study design. I was especially impressed with the detour the driver took to ensure the travel time of the rats from the breeding facility to their new rearing environment was standardized. Just to confirm, all the pregnant mothers came from the same stock from Janvier Labs, does this mean that all mothers also came from the same colony room? I assume so, but this should be made clearer in the methods. For replication purposes, if animals came from multiple rooms at Janvier labs, it should be indicated which rooms, and how many dams from each room went to each rearing site. This is because there appear to be phenotypic differences across rooms in commercial facilities, not only across different breeding sites. Similarly, were all animals (dams and offspring) housed in the same colony room at each rearing site, or were males and females housed in different rooms, for example?

Thank you for your positive feedback on our effort to design this study in a meaningful way to tease apart genetic and other environmental factors from the role of the rearing environment. Yes, indeed all time-mated pregnant females came from the same colony room (more precisely room A03) at Janvier Labs. To exclude confounding by other environmental factors, we specifically requested that all animals come from the same breeding room. We have clarified this in the revised manuscript (Line 360).

Housing conditions varied among the participating laboratories – this includes among other factors co-housing of males and females in the same room. As we were specifically interested in the extent to which common variation in local housing and husbandry conditions during rearing will affect the animals' phenotype, we did not want the rearing labs to harmonize housing conditions, but deliberately asked them to house the animals according to their standard laboratory practice.

All housing and husbandry related information for each of the five rearing facilities and the testing laboratory were presented in the supporting table 10. As requested by the Reviewer, in the revised version we now cite this table as Supporting Table 1 (S1 Table) in the main text (Lines 93-96).

**2.** I may have misunderstood how it was written in the methods, but it seems there were at least some cases where multiple same-sex pups from a single litter were used on a measure. If so, how were litter effects controlled for? The authors should make a table showing how many litters (and how many total of each male and female offspring) from each of the five rearing environments were represented on each endpoint of interest.

We have tried to clarify this by adding a supporting figure (S6 Figure) describing our weaning strategy (which was standardized across all rearing facilities) in detail. In addition, we describe in the text (Lines 370-375) for which outcome measures we had used one or two same-sex littermates, respectively. Litter effects were controlled for by including mice nested in cage as a factor in the analysis whenever two same-sex littermates were used.

There were only three outcome measures at TP2, for which both remaining littermates/cagemates were used (body weight, adrenal gland weight, and microbiome composition). This allowed us to analyse within vs. between litter/cage variation for some variables, which was particularly relevant for the microbiome composition analysis. For all other outcome measures, we used only one mouse per litter/cage, whereby at TP2 one littermate was allocated to behavioral testing while the other one remained test-naïve and was used for chromatin profiling. This was the same for all five rearing facilities, so there is no variation between rearing facilities caused by litter effects.

**3.** The authors report that variation in litter size did not impact behavior. Litter size was not standardized either within or across rearing environments. The authors should provide a table showing the range of litter sizes across each of the rearing environments. Were they comparable across sites? Litter size prior to weaning can impact later life body weight, and this could affect differences between stress physiology (indeed basal cort was different as a function of litter size), microbiome, and behavior. Can the authors test if litter size acts as a moderating variable between body weight and the other endpoints of interest?.

Variation in both litter size and culling (see e.g. Mendi 1988, Palmer and Ulbrich 1997, Laviola 1995, Suvorov and Vandenberg 2016) can affect physiology and behavior. Therefore, we decided not to standardize (i.e. cull) litters but instead to include both litter size and litter sex ratio in our statistical model. As shown in Supporting Table 4, the rearing facility (RF) was the only factor that had a strong and persistent effect on body weight. Since litter size had no significant effect on body weight, it was unlikely to act as a moderating variable and influence other outcome variables.

As requested by the Reviewer, we added a new supporting table (S11 Table), which shows litter size within and across rearing facilities. A graphical representation of variation in litter size is shown below. Based on the Reviewer's comment, we tested if litter size varied across rearing facilities and found that rearing facility had a small effect on litter size in males (p=0.044; without correction for multiple testing) but not in females (p=0.137).

| Sex | Variables | Df | Sum Sq | Mean Sq | Adjusted  R-squared | F value | p |
| --- | --- | --- | --- | --- | --- | --- | --- |
| Males | Rearing Facility | 4 | 23.233 | 5.8083 | 0.09918 | 2.6239 | 0.04437* |
|  | Residuals | 55 | 121.750 | 2.2136 |  |  |  |
| Females | Rearing Facility | 4 | 17.433 | 4.3583 | 0.05291 | 1.824 | 0.137 |
|  | Residuals | 55 | 131.417 | 2.3894 |  |  |  |

**4.** Gut microbiome changes have a known influence on social behavior and pain sensitivity. Did the authors look at any behavioral tests evaluating these, or other tests evaluating cognitive measures?

Our study was designed to probe effects of the rearing facility on a broad spectrum of outcome variables, including morphological, physiological and behavioural variables, microbiota and the epigenome, with a specific focus on measures of anxiety and stress. This meant that we had to be selective, and due to the size of the study, we had to use relatively simple behavioural tests that did not require extensive habituation and training. We agree that given the variation we observed in the gut microbiome, it would be interesting to study whether this is associated with variation in social behavior, pain sensitivity, and cognitive measures, and we hope that our study will stimulate others to address these effects.

**5.** Supplementary Tables 10a and 10b are placed towards the end of the supplementary materials. However, these tables contain many important methodological details that answered several questions I initially had. I suggest these two tables be moved from supplementary and into the main paper.

We have moved the supporting table 10 into the main text (now S1 Table).

**Reviewer #2:**
[identifies herself as Vanessa Beijamini]
Jaric and colleagues investigated the influence of common differences in the rearing environment on behavior, gut microbial community, and epigenetic of male and female adult inbred mice. They proposed an important hypothesis and an interesting experimental design to test it, controlling the mice genotype and test conditions. The work is clearly and accurately presented, with sufficient details of methods and results. The experimental design is appropriated to test the hypothesis. The conclusions drawn were adequately supported by the results. Notwithstanding, the authors followed very good practices to increase reproducibility (detailed methods, study pre-registration, sample size calculation, randomization, blindness of experimenters, provision of additional data, and detailed statistical analysis).

Thank you very much for this positive feedback.

**Minor Issues:
1.** Why were data from male and female mice analyzed separately?

This was a difficult decision that we had taken based on the following considerations. First, we were primarily interested in assessing the extent to which rearing facility affects the phenotype of mice, and for this, we wanted to include both males and females. However, we were not specifically interested in directly comparing the size of effects between males and females. Second, co-housing males and females in open cage systems (as was the case in our testing laboratory) may enhance aggression among male mice. We therefore decided to house males and females in separate housing rooms. This in turn meant that a direct comparison between males and females was no longer possible, because sex would have been confounded with housing room (see also comment 1 by reviewer 1). Therefore, males and females had to be analysed separately.

2.      In the light-dark box, was there a delay between releasing the animal into the dark compartment and opening the door to avoid entry into the light compartment due to escape motivation from the experimenter?

Yes, we have clarified this in the methods section, which now reads as follows (Lines: 542-543): *"Each mouse was placed in the dark compartment of the apparatus, and testing began after a delay of 5 seconds during which the sliding door to the light compartment was raised. Test duration was 10 minutes."*

**Reviewer #3:**
In this manuscript, the authors described a comprehensive study on how environmental differences between animal facilities affect mouse phenotypes. The authors randomly allocated pregnant mice from the same breeding cohort to five different facilities and analyzed the phenotypes of progenies before and after transportation to a single test laboratory. They found the gut microbiomes had different compositions and heterogeneity, liked caused by the differences in diets. Mice from different facilities also showed different body weights and behavioral phenotypes. They mapped the chromatin accessibility in the ventral hippocampus by ATAC-seq and detected differences in genes involved in synaptic plasticity, neurogenesis and signaling pathways. The authors proposed that animal studies should be done across facilities to increase the robustness and replicability of findings.

Please find below several questions and suggestions that I hope will improve the manuscript.

**1.** The authors reported several phenotypic and molecular differences in mice from different facilities. It would be very helpful if the authors could study or discuss whether these phenotypic and molecular differences are connected to help reveal the underlying mechanisms. For example, does the difference in microbiome composition drive the difference in weight gain and behavior phenotypes? The authors could transfer fecal contents of mice from different facilities into germ-free mice and see if the microbiota causally causes differences in weight, behavior, or neural chromatin structures. The authors may cohouse the mice from different facilities or treat them with antibiotics to see if the gut microbiome drives these phenotypical and molecular differences.

We agree that there is a need to study these connections further to reveal the underlying mechanisms. However, the suggestions made by the reviewer go beyond the scope of this study. As explained above in response to comment 4 by Reviewer 2, our study was designed to assess effects of common variation among housing and husbandry conditions between rearing facility on a broad spectrum of phenotypic outcome variables, including morphological, physiological and behavioural variables, microbiota and the epigenome. Furthermore, due to the largely exploratory nature of the study, it would not have been appropriate to test specific hypotheses derived from our findings using the same data. Nevertheless, we added a paragraph to the discussion section (Lines 322-331), discussing possible underlying mechanisms that would be consistent with our data, and highlighting the need for further hypothesis-driven research in this direction. We hope that our study will stimulate such further work, and will certainly follow-up on some of these ideas in our own future work.

**2.** The authors suggested that the difference in diet drives the difference in the gut microbiome. The authors may use two different diets to confirm whether diet drives phenotypic and molecular differences when mice are housed in the same facility. A related question is that about 20% of variances in microbiome and behaviors can be explained by the difference in animal facilities. While the authors listed some other factors such as litter size, litter sex ratio and group size, how do these factors increase/affect explainable variances? The result will help evaluate the significance of animal facilities in driving phenotypic variation.

We agree with the reviewer that our finding of an association between differences in diet and differences in the gut microbiome needs independent experimental confirmation, which was however beyond the scope of this study.

The proportion of variance explained by other factors can be approximately derived from the Pillai statistic given in the Manova table S5 (see Table below). Rearing facility explains approximately 10 times (for males) or 12 times (for females) as much of the total variation than sex ratio, and between rearing facility and litter size we see a four-fold difference in the explained variance. In females, rearing lab explains also approximately twice as much of the overall behavioural variation than estrous cycle stage. After reanalysing these results, however, we have to admit that we discovered a mistake in the originally reported results that we have now corrected. Thus, the proportions of the total behavioural variance explained by rearing facility are 14.4% for males and 12.4% for females, and the partial variance components (now added to the text; Lines 164-167) are 23% for males and 25% for females. Proportions were corrected in the table and in the text; test statistics and p-vales were not affected and remained unchanged.

| Sex | Variables | Pillai | Eta^2 | %Var |
| --- | --- | --- | --- | --- |
| Males | Rearing Facility | 0.86346 | 0.144 | 14.4 |
|  | Litter size before weaning | 0.14918 | 0.025 | 2.5 |
|  | Sex ratio | 0.08369 | 0.014 | 1.4 |
|  | No of cage mates after weaning | 0.17010 | 0.028 | 2.8 |
| Females | Rearing Facility | 0.74167 | 0.124 | 12.4 |
|  | Litter size before weaning | 0.17471 | 0.029 | 2.9 |
|  | Sex ratio | 0.05987 | 0.010 | 1.0 |
|  | No of cage mates after weaning | 0.06705 | 0.011 | 1.1 |
|  | OF ECS | 0.40478 | 0.067 | 6.7 |
|  | LDB ECS | 0.50042 | 0.083 | 8.3 |

**3.** In Fig 3c, there are some big changes in chromatin accessibility between TP1 and TP2. What are the genes that are regulated by these peaks? Technically, it is unclear how the authors defined the most and least accessible sites.

We added a description in the method section (Lines : 714-716) *"Within each sample, the most and least accessible sites were defined as the peaks with the 2.5 % highest and 2.5 % lowest sequence counts. Sites from all samples and time points were merged to generate the heatmap shown in Figure 3c*". Given the simple definition of these sites, we did not discuss the underlying genes. To compare the two time points to each other, the differential binding analysis would be more appropriate/powerful. However, we decided against the direct comparison because the data of the two time points were derived from two separate sequencing batches of mice.

**4.** In several places, figure references are mislabeled. For example, line 182 should be Fig 2c instead of fig 1c. Line 185, where are Fig1 g,h. On line 219, where is Fig. 2g? Should be a supplemental figure.

Thank you for noticing these. We have corrected them in the revised version.

**5.** PND should be defined in figure 2 legends.

We have defined PND as suggested.
